# Bayesian benchmark dose modeling analysis and derivation of points of departure for female reproductive toxicity following exposure to di(2-ethylhexyl) phthalate (DEHP) — effects on reproductive hormones, folliculogenesis and estrous cyclicity

**DOI:** 10.1093/toxsci/kfaf052

**Published:** 2025-04-21

**Authors:** Antero Vieira Silva, Ilari Tarvainen, Mattias Öberg, Mary Laws, Patrick Hannon, Jodi Flaws, Pauliina Damdimopoulou

**Affiliations:** Unit of Integrative Toxicology, Institute of Environmental Medicine, Karolinska Institutet, Stockholm 171 65, Sweden; Department of Women’s and Children’s Health, BioMedicum, Karolinska Institutet, Stockholm 171 65, Sweden; Department of Obstetrics and Gynecology, University of Helsinki, Helsinki University Central Hospital, Helsinki 00290, Finland; Unit of Integrative Toxicology, Institute of Environmental Medicine, Karolinska Institutet, Stockholm 171 65, Sweden; Department of Comparative Biosciences, University of Illinois Urbana-Champaign, Urbana, IL 61802, United States; Department of Obstetrics & Gynecology, College of Medicine, University of Kentucky, Lexington, KY 40508, United States; Department of Comparative Biosciences, University of Illinois Urbana-Champaign, Urbana, IL 61802, United States; Carl R. Woese Institute for Genomic Biology, University of Illinois Urbana-Champaign, Urbana, IL 61801, United States; Department of Women’s and Children’s Health, BioMedicum, Karolinska Institutet, Stockholm 171 65, Sweden; Department of Gynecology and Reproductive Medicine, Karolinska University Hospital, Stockholm 141 57, Sweden

**Keywords:** benchmark dose (BMD), di(2-ethylhexyl) phthalate (DEHP), endocrine disrupting chemicals, estrous cyclicity, folliculogenesis, hypothalamus–pituitary–gonadal axis, ovarian follicles, reproductive toxicity

## Abstract

Endocrine-disrupting chemicals such as di(2-ethylhexyl) phthalate (DEHP) pose significant risks to human reproductive health. However, regulatory frameworks often lack sufficient data on sensitive female-specific reproductive endpoints. This study investigates the sensitivity of hypothalamic–pituitary–ovarian (HPO) axis endpoints to DEHP exposure in adult female mice, applying Bayesian Benchmark dose (BBMD) modeling for dose–response assessment and derivation of points-of-departure (PODs) for risk assessment. Data from four studies where sexually mature female mice were exposed to DEHP (0.02 to 240 mg/kg bw/d) for 10 or 30 d via oral administration, or 30 d via diet, was modeled. Endpoints included ovarian follicle counts, serum hormones, estrous cyclicity, body, and organ weights. Results revealed dose-dependent changes and greater sensitivity of progesterone, ovarian follicle counts, and uterine weight, compared with estrous cyclicity, body weight, and other organ weights. For 10- and 30-d oral administration studies, the lowest nonzero BBMDLs were observed for serum progesterone levels (9.1 mg/kg bw/d) and primary follicle counts (19.5 mg/kg bw/d), respectively. These PODs were notably lower than most No-Adverse-Effect-Levels in the European Chemicals Agency’s (ECHA’s) “Registered substances factsheet” and “ECHA CHEM” databases. The majority of the studies derived PODs based on male (reproductive) endpoints. Finally, a derived no-effect level of 0.064 mg DEHP/kg bw/d was estimated, based on the overall lowest BBMDL, serum progesterone levels of the 10-d oral study. In conclusion, our study indicates that current guidelines may not fully capture reproductive risks for females, underscoring the need to refine regulatory endpoints to better protect female reproductive health in the context of DEHP exposure.

Reproductive toxicity assessment is a vital component of chemical regulatory frameworks, involving multiple approaches such as tiered testing, standardized animal testing, endocrine disruptor screening, and developmental neurotoxicity testing, ultimately resulting in a globally harmonized system (GHS) classification for reproductive toxicants (Piersma et al. 2007). Many of these testing methodologies are standardized by the Organization for Economic Cooperation and Development (OECD), covering reproductive endpoints in single or multigenerational studies (Piersma et al. 2007; OECD 2018a). A tiered testing strategy is frequently used, as in the European Union’s REACH regulation (Regulation (EC) No 1907/2006), beginning with screening studies and progressing to more comprehensive evaluations based on initial findings (European Chemicals Agency (ECHA) 2021b). A weight-of-evidence approach incorporates data from multiple studies and endpoints for hazard assessment, aiming to effectively identify reproductive hazards, while minimizing animal use and optimizing resource allocation in regulatory toxicology (European Chemical Agency (ECHA) 2016). Finally, regulatory frameworks typically estimate chemical exposure limits to prevent adverse effects on humans and the environment, but may fall short due to insufficient data on relevant endpoints.

The OECD and the ECHA, together with the European Food Safety Authority (EFSA), have comprehensive frameworks for testing endocrine-disrupting compounds (EDCs), aiming to standardize the identification and assessment of substances that may interfere with hormonal systems (European Chemical Agency (ECHA) and European Food Safety Authority (EFSA) with the Technical Support of the Joint Research Centre (JRC) et al. 2018; OECD 2018a). Both frameworks emphasize the importance of considering endpoints from multiple endocrine pathways, such as estrogenic, androgenic, thyroid, and steroidogenic pathways. Moreover, it is recommended to assess a combination of mechanistic and apical endpoints to ensure a thorough and consistent evaluation of the chemical’s endocrine-disrupting potential in intact organisms (European Chemical Agency (ECHA) and European Food Safety Authority (EFSA) with the Technical Support of the Joint Research Centre (JRC) et al. 2018; OECD 2018a).

Phthalates are synthetic anthropogenic chemicals widely used in consumer products, medical devices, and plastics (Kay et al. 2013). These semipersistent chemicals contribute to environmental contamination both as parent compounds and as metabolized downstream products, which often exhibit higher bioactivity than the parent compounds (Kay et al. 2013). These metabolites have been detected in human serum, urine, saliva, breast milk, and follicular fluid (Hines et al. 2009; Krotz et al. 2012; Du et al. 2016; Bellavia et al. 2023). Studies have shown that individual phthalates and phthalate mixtures can disrupt both testicular and ovarian gene expression and steroidogenesis *in vivo* and *in vitro* (Lovekamp and Davis 2001; Gupta et al. 2010; Zhou and Flaws 2017; Chiang et al. 2020a; Tarvainen et al. 2023). Additional effects on female reproduction include decreased mouse antral follicle growth, oocyte fragmentation, and disrupted steroidogenic enzyme expression in the F0 generation, as well as transgenerational effects on reproductive toxicity in the F1 generation (Moyer and Hixon 2012; Zhou et al. 2017).

Di(2-ethylhexyl) phthalate (DEHP, CAS number 117-81-7) has been classified as GHS reproductive toxicant category 1B under the Classification, Labelling and Packaging (CLP) regulation, and is considered an EDC, and presumed to be a human reproductive toxicant based on strong evidence from animal studies (European Parliament and Council 2008; European Chemical Agency (ECHA) and European Food Safety Authority (EFSA) with the Technical Support of the Joint Research Centre (JRC) et al. 2018). The findings in these studies were predominately derived from studies using male rats. However, DEHP has also been linked to reproductive toxicity in females, including an increased risk of decreased ovarian reserve and disruption of gonadotropin-dependent follicle growth in both women and mice (Hannon et al. 2014; Hannon and Flaws 2015; Messerlian et al. 2016; Chiang and Flaws 2019; Bellavia et al. 2023; Li et al. 2024). A recent study on women undergoing infertility treatments sheds light on potential mechanisms, showing that follicles with higher DEHP metabolite levels respond less to hormone stimulation and have reduced cholesterol biosynthesis, decreased steroidogenesis, and increased inflammation compared with follicles in the lowest quartile of exposure (Varik et al. 2024). These human data support the classification of DEHP as a reproductive toxicant and EDC in females.

Despite growing evidence of the adverse effects of DEHP on females, risk assessments specifically addressing female reproductive endpoints have not been conducted. As a result, it remains unclear whether guidance values established based on male data are equally protective for females. Previous *in vivo* studies have shown that even short-term exposure to DEHP can affect fertility and reproductive endpoints both acutely and after extended recovery periods (Hannon et al. 2016; Chiang et al. 2020a, 2020b; Laws et al. 2023; Safar et al. 2023; Santacruz-Márquez et al. 2024). Given the long reproductive lifespan in humans and the continuous, lifelong exposure to compounds such as DEHP, it is essential to reliably assess the risks to female fertility. The remaining critical knowledge gaps include the identification of sensitive fecundity endpoints for these assessments. For example, the relative sensitivities of classical female reproductive toxicity endpoints such as estrous cyclicity, ovarian steroidogenesis, and folliculogenesis have not been thoroughly investigated. Identifying the critical endpoints is crucial for improving chemical health risk assessments for female reproductive toxicity.

The regulation of female fertility in adulthood is an intricate process governed by complex feedback loops along the hypothalamus–pituitary–ovarian (HPO) axis. This system controls the cyclical modulation of various hormones during the reproductive cycle such as gonadotropin-releasing hormone, follicle-stimulating hormone (FSH), and luteinizing hormone (LH), which are secreted by the hypothalamus and pituitary glands to direct the maturation of ovarian follicles and trigger ovulation (Canipari et al. 2020). Ovarian follicles, in turn, act as a central site for the generation of sex steroid hormones such as progesterone and estradiol, which regulate the cyclical patterns of female fertility and uterine receptivity for embryo implantation and contribute to both positive and negative feedback mechanisms in the HPO axis (Canipari et al. 2020). Ovarian follicles form prenatally in humans and perinatally in mice and rats. The growth of these primordial follicles containing immature oocytes to ovulation can be divided into gonadotropin-independent and -dependent growth phases that together take multiple estrous cycles. Follicles grow from dormant primordial stage through granulosa cell proliferation to primary, pre-antral, and antral stages within the ovaries. Although follicles can initiate growth even before puberty, they can only complete their maturation after puberty. Throughout a woman’s reproductive life, the controlled periodic fluctuations in hormone levels shape reproductive health, fecundity, and the aging process (Reed and Carr 2000). Disruption of follicular development or hormonal homeostasis can lead to adverse outcomes such as infertility, premature reproductive senescence, and sterility (Silva et al. 2023). In regulatory toxicity assessments, the impact of chemicals on these processes is evaluated by assessing ovarian function (folliculogenesis and ovulation), hormonal balance (progesterone, estradiol levels, LH, and FSH), estrous cycle length and regularity, mating and fertility rates, gestation length and birth outcomes, among other endpoints (US EPA 1996; Silva et al. 2023).

This study addressed knowledge gaps regarding the sensitivity of common female reproductive toxicity endpoints in adult animals. Bayesian benchmark dose (BBMD) modeling was used to assess the short-term, dose-dependent effects of DEHP on serum steroids, ovarian follicle populations, organ weights, and estrous cyclicity in adult female mice. The derived points-of-departure (PODs) were then compared with No-Adverse-Effect-Level (NOAEL) values from (key) DEHP studies reported by registrants. Our study highlights the varying sensitivity of female reproductive toxicity endpoints, particularly underscoring follicle counts and serum progesterone as highly sensitive indicators, with PODs comparable to or lower than many regulatory NOAELs.

## Materials and methods

### Selection of studies

Studies selected for BMD analysis exposed sexually mature adult female mice to DEHP, either orally (10 or 30 d) (Hannon et al. 2014; Chiang and Flaws 2019) or in the diet (30 d) (Laws et al. 2023; Safar et al. 2023; Santacruz-Márquez et al. 2024). In all of these repeated dose toxicity studies, sexually mature female mice were exposed to standardized doses ranging from 0.02 to 240 mg/kg per body weight per day, and similar female reproductive toxicity endpoints were measured. Raw data were available for all animals. All studies were performed at the University of Illinois at Urbana-Champaign in the same laboratory, under the same conditions, during the period 2014 to 2023, as explained below.

### Animals

In all selected studies, adult CD-1 female mice were purchased from Charles River Laboratories (Wilmington, MA) and acclimatized for up to 40 d before the study start. Table 1 summarizes the study design. Mice were given *ad libitum* access to purified water treated by reverse osmosis-treated and Teklad Rodent Diet (8604). Facilities were maintained at 21°C ± 2°C, with humidity levels set at 50%±20%, and a 12-h light-dark cycle. All procedures and animal handling were approved by the University of Illinois at Urbana-Champaign Institutional Animal Care and Use Committee.

**Table 1.** Overview of the four selected studies analyzing the effects of short-term exposure to DEHP on female reproductive toxicity endpoints.

*Study*	*Reference*	*Doses*	*Group size*	*Endpoints measured*
10-d oral exposure, study I	Hannon et al. (2014)	0, 0.02, 0.2, 20, and 200 mg/kg bw/d	*n *= 8 per dose group, female mice only	Estrous cyclicity, serum hormones, and ovarian follicle number
10-d oral exposure, study II	Chiang and Flaws (2019)	0, 0.02, 0.2, 20, and 200 mg/kg bw/d	*n *= 6 per dose group, female mice only	Body and organ weights
30-d oral exposure	Hannon et al. (2014)	0, 0.02, 0.2, 20, and 200 mg/kg bw/d	*n *= 8 per dose group, female mice only	Estrous cyclicity, serum hormones, and ovarian follicle numbers
30-d dietary exposure	Laws et al. (2023), Safar et al. (2023), Santacruz-Márquez et al. (2024)	0, 0.024, 0.24, 24, and 240 mg/kg bw/d	*n *= 10 per dose group, female mice only	Estrous cyclicity, serum hormones, and ovarian follicle numbers

Some studies were published across multiple papers; for the present study, original raw data were used for modeling.

### Chemicals and dosing

The same source of DEHP was used in all four studies (Sigma-Aldrich, catalog number D201154, St Louis, MO), and the doses were chosen based on the U.S. Environmental Protection Agency (EPA) reference dose for oral exposure (0.02 mg DEHP/kg/d) and the estimated range of human daily exposure to DEHP (3 to 30 mg/kg/d) (Doull et al. 1999; US EPA 2000). The 200-mg DEHP/kg/d dose aimed at the median of the estimated occupational exposure range for occupational exposure (143 to 286 mg/kg/d) (Kavlock et al. 2002).

For the oral exposure studies, stock solutions in corn oil were diluted in serial dilutions, from the highest concentration to the working doses of 200, 20, 0.2, and 0.02 mg DEHP/kg/bw/d. The final dosing volume was determined daily based on the mouse’s body weight. The dose was given by inserting the pipette tip beyond the incisor teeth, toward the cheek pouch. For the dietary study, DEHP was incorporated in the chow by Envigo Teklad Diets (Madison, WI), and was given *ad libitum*. Assuming an average mouse body weight of 25 g and a daily consumption of ∼5 g of chow, the target dietary concentrations were 1,500, 1.5, and 0.15 ppm DEHP, resulting in estimated exposures of ∼240, 0.24, and 0.024 mg DEHP/kg body weight per day.

### Endpoints

Reproductive toxicity endpoints were analyzed in all euthanized female mice, with a focus on typical OECD guideline assay endpoints, as outlined in Table 2. In short, chosen endpoints were of regulatory relevance, including estrous cyclicity, serum hormones, ovarian follicle counts, body, and organ weights (absolute and relative to final body weight). Animals were euthanized in estrus (Hannon et al. 2014) or diestrus (Chiang and Flaws 2019; Laws et al. 2023; Safar et al. 2023; Santacruz-Márquez et al. 2024). Details on estrous cyclicity profiling, serum hormone measurements, ovarian histology, body, and organ weight measurements are shown in Table 2 and further information is given in the original studies (Hannon et al. 2014; Chiang and Flaws 2019; Laws et al. 2023; Safar et al. 2023; Santacruz-Márquez et al. 2024).

**Table 2.** Detailed overview of the reproductive toxicity endpoints, their assessment methods, and outcome variables used in the BBMD analysis.

***Estrous cyclicity*** (Hannon et al. 2014; Laws et al. 2023)	
*Method of assessment*	*Outcome variable*
Vaginal lavage sample examined for cytology under a light microscope, using previously defined criteria (Goldman et al. 2007)	% days in estrus % days in metestrus/diestrus

***Serum hormones*** (Hannon et al. 2014; Safar et al. 2023; Santacruz-Márquez et al. 2024)	
*Method of assessment*	*Outcome variable*
Enzyme-linked immunosorbent assay (ELISA)	Serum progesterone (ng/ml) Serum estradiol (pg/ml)

***Ovarian follicles*** (Hannon et al. 2014; Laws et al. 2023; Safar et al. 2023; Santacruz-Márquez et al. 2024)	
*Method of assessment*	*Outcome variable*
Manual or automated count (using the NDP.view2 software [Hamamatsu Photonics K.K.]) in every 10th section of the fixed entire ovaries, examined in a glass slide under a light microscope, using previously defined criteria (Hannon et al. 2014).	Total follicle count Primordial follicles, total Primary follicles, total Preantral follicles, total Antral follicles, total Early follicles (primordial+primary), total Late follicles (antral+preantral), total

***Body and organ weights*** (Chiang and Flaws 2019)	
*Method of assessment*	*Outcome variable*
Manual weighing after euthanasia. Relative weights were calculated by dividing the organ weight by the body weight after euthanasia.	Initial weight, g Final body weight, g Body weight change, % Ovary weight, absolute and relative Uterine weight, absolute and relative Liver, absolute and relative

### Bayesian benchmark dose modeling, selection of the critical effect size, and quality control criteria

A BBMD framework (Shao and Shapiro 2018) was employed for dose–response assessment, which is publicly available at https://www.benchmarkdose.org (maintained by DreamTeach LLC). This full Bayesian dose–response approach employs the Markov Chain Monte Carlo (MCMC) algorithm and Bridge Sampling to estimate parameters and determine the credible interval for benchmark doses, including the lower (BBMDL) and upper (BBMDU) bounds of the 90% credible interval. Additionally, BBMD performs Bayesian model averaging to address model uncertainty across individual models fitted to the data.

For the dose–response analysis, raw individual data from the original four studies were kindly provided by the authors. The data were assumed to be log-normally distributed. One MCMC with 30,000 iterations was run, with a 50% warm-up rate. An uninformative prior was chosen, reflecting an equal probability of the BMD across the possible range. Five continuous models were fitted to the data (Hill, Power, Michaelis–Menten, Linear, and the best-fitting Exponential 3 or 5). The model weight prior was left unchanged at 0.2, as BBMD adjusts the posterior model weights according to the model fit.

The critical effect size (CES), also referred to as the Benchmark response, is by definition the threshold for adversity, above which effects should be considered adverse. Expert judgment established a CES of ±5% for body and organ weights, and ±10% for HPO endpoints, in relation to the estimated background, using the central tendency BMD estimation method. If the resulting BMDL was ≤0, due to the possibility of the Linear and Michaelis–Menten models resulting in *x *≤ 0 when *y *= 0, the BMDL was replaced by a zero.

The independent variable was the DEHP external dose. In all studies, the exposures ranged from 0.02 to 240 mg DEHP/kg bw/d, with four dose groups and the control group (Table 1). The dependent variables were HPO axis endpoints: Estrous cyclicity, serum hormones, ovarian follicle counts, and body and organ weights (Table 2).

The resulting lower (BMDL) and upper bound (BMDU) of the 90% credible interval, as well as the benchmark dose (BMD) were assessed using quality control (QC) criteria as recommended by EFSA (2022). Specifically, the QC exclusion criteria used were: BMD/BMDL>20, BMDL 10 times lower than the lowest nonzero dose (i.e. 0.002 mg DEHP/kg bw/d) or BMDU/BMDL>50.

### Dossier data

**Fig. 1.**
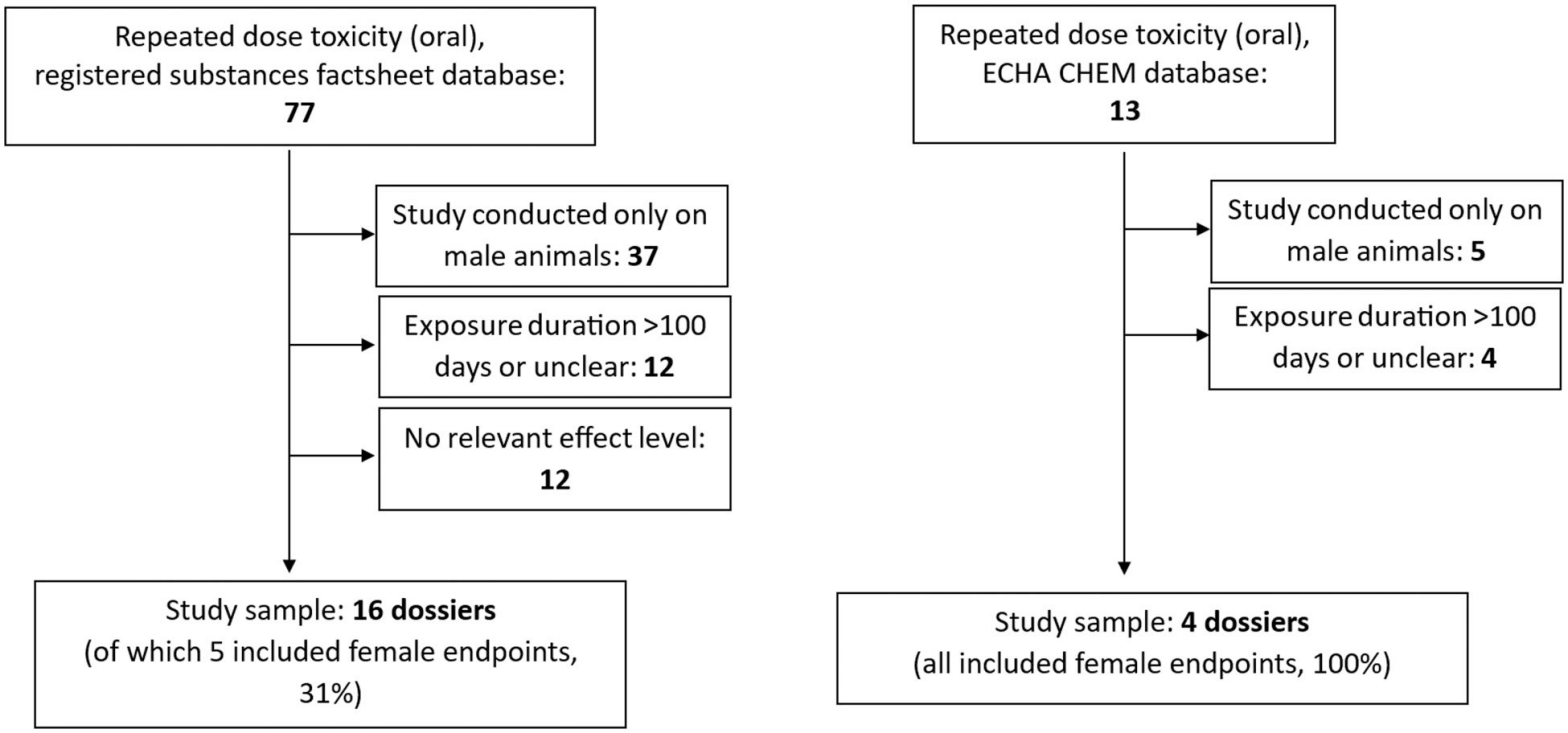
Flowchart diagram of the study selection process for the repeated dose toxicity studies, for the “Registered substances factsheet” and “ECHA CHEM” databases. Figures indicate the total number of studies in each step.

**Fig. 2.**
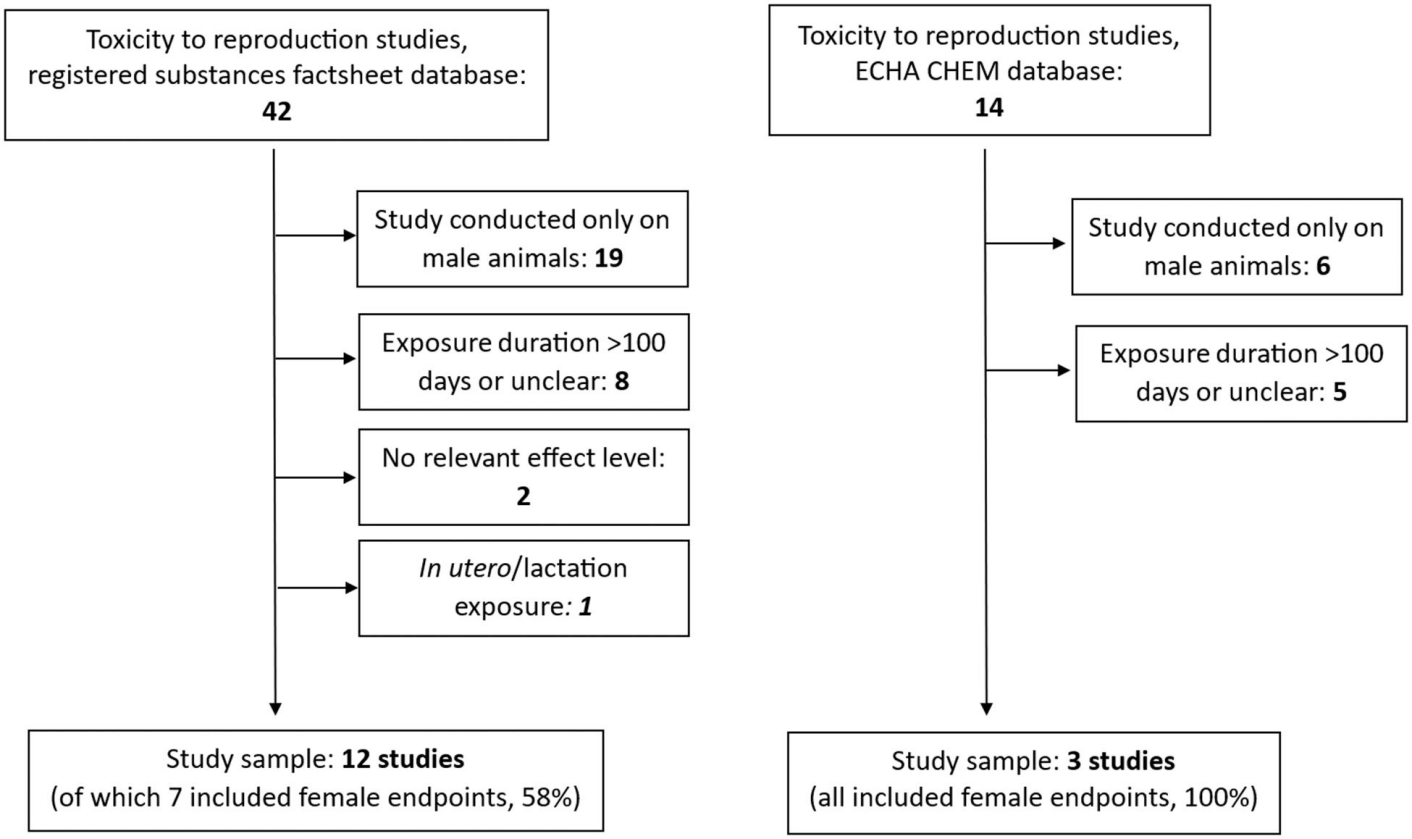
Flowchart diagram of the study selection process for the toxicity to reproduction studies, for the “Registered substances factsheet” and “ECHA CHEM” databases. Figures indicate the total number of studies in each step.

Data from the full registration dossier of DEHP, submitted under the REACH Regulation (EC 1907/2006), were retrieved. This included information from both the previous public “Registered substances factsheet” database on the ECHA website (echa.europa.eu; accessed: September 10, 2024 [database not updated since May 19, 2023]) and the new “ECHA CHEM” database launched in January 2024 (chem.echa.europa.eu; accessed: September 24, 2024) registration dossier (Figs 1 and 2). The derived no-effect level (DNEL) estimated by the registrant was also extracted. Data on repeated dose toxicity (oral exposure) and reproductive toxicity were collected, including species, study duration, and relevant search terms for female reproductive endpoints (e.g. follic*, ovar*, proges*, uter*, and estrus*), as well as NOAEL or LOAEL values from the studies, and criteria for selecting critical endpoints.

Exclusion criteria were applied to studies if: (i) the involved only male animals, or (ii) the study duration exceeded 100 d or was unspecified, or (iii) NOAEL/LOAEL values were not reported in mg/kg bw/d. During the transition to ECHA CHEM, studies may have been recategorized or renumbered. To avoid duplication, study details were compared across platforms to ensure each study was included only once. However, if a repeated dose toxicity study provided a NOAEL based on reproductive toxicity endpoints, it was included in both result sections.

## Results

To investigate the relative sensitivity of standard guideline-based endpoints for female reproductive toxicity, we extracted raw data on estrous cycles, serum hormone levels, follicle counts, and organ weights from four original studies involving DEHP exposure in adult mice (Tables 1 and 2). These data were used to derive BMDLs, BMDs, and BMDUs, enabling comparisons across endpoints. Results for HPO axis endpoints are presented in Table 3, and organ weights are provided in Table 4. Models that met EFSA’s QC criteria for BMD are highlighted in bold, and for each study, the two most sensitive endpoints, indicated by the lowest BMDL and BMD values, are underlined.

**Table 3.** HPO endpoints (estrous cyclicity, serum hormones, and ovarian follicles) were analyzed using Bayesian benchmark dose–response analysis, following DEHP oral exposure for 30 d either orally (Hannon et al. 2014) or via the diet (Laws et al. 2023; Safar et al. 2023; Santacruz-Márquez et al. 2024).

	**10-d oral exposure, study I** (Hannon et al. 2014)						**30-d oral exposure study** (Hannon et al. 2014)						**30-d dietary exposure study** (Laws et al. 2023; Safar et al. 2023; Santacruz-Márquez et al. 2024)					
	CES	BMDL	BMD	BMDU	BMD/BMDL	BMDU/BMDL	CES	BMDL	BMD	BMDU	BMD/BMDL	BMDU/BMDL	CES	BMDL	BMD	BMDU	BMD/BMDL	BMDU/BMDL
***Estrous cyclicity***																		
% days in estrus	+10%	0	230	1,329	NA	NA	+10%	**97.2**	**206**	**949**	2.1	9.8	+10%	**176**	**234**	**366**	1.3	2.1
% days in metestrus/diestrus	−10%	**134**	**240**	**1,582**	1.8	11.8	−10%	**90.3**	**181**	**607**	2.0	6.7	−10%	**246**	**289**	**638**	1.2	2.6
***Serum hormones***																		
Serum progesterone (ng/ml)	+10%	**9.1**	**36.6**	**204**	4.0	22.4	−10%	**26**	**108**	**797**	4.1	30.6	−10%	0	44.2	314	NA	NA
Serum estradiol (pg/ml)	+10%	0	85.4	318	NA	NA	−10%	**48**	**109**	**394**	2.3	8.3	−10%	**55.1**	**203**	**1,118**	3.7	20.3
***Ovarian follicles***																		
Total follicle count	−10%	**75.9**	**193**	**1,227**	2.5	16.2	−10%	0	198	1,206	NA	NA	+10%	**43.3**	**165**	**740**	3.8	17.1
Primordial follicles, total	−10%	2.6	193	1,499	74.0	576.7	−10%	0	178	1,114	NA	NA	+10%	**23.2**	**128**	**742**	5.5	32.0
Primary follicles, total	+10%	**19.5**	**31**	**172**	1.6	8.8	+10%	**105**	**275**	**2,754**	2.6	26.3	+10%	**50.8**	**221**	**1,310**	4.4	25.8
Preantral follicles, total	−10%	**142**	**193**	**284**	1.4	2.0	−10%	**37.5**	**97**	**264**	2.6	7.1	+10%	**42.2**	**161**	**681**	3.8	16.1
Antral follicles, total	−10%	0	103	392	NA	NA	−10%	**48.4**	**105**	**283**	2.2	5.8	+10%	0	37.8	239	NA	NA
Early follicles (primordial and primary), total	−10%	**51.7**	**177**	**992**	3.4	19.2	−10%	**176**	**211**	**364**	1.2	2.1	+10%	0	113	613	NA	NA
Late follicles (antral and preantral), total	−10%	0	166	748	NA	NA	−10%	**60.5**	**124**	**341**	2.1	5.6	+10%	**39.5**	**132**	**490**	3.3	12.4

The BMDL, BMD, and BMDU units are mg DEHP/kg bw/d. A positive CES denotes an upward/increasing dose–response, whereas a negative CES a downward/decreasing dose–response relationship. Bolded figures highlight the accepted models according to the employed EFSA QC criteria, and the two lowest BMDLs and BMDs per study are underlined. NDR, no dose–response; NA, not available/not possible to estimate.

**Table 4.** Body and organ weight endpoints were analyzed using Bayesian benchmark dose–response analysis, following DEHP oral exposure for 10 d (Chiang and Flaws 2019).

	**10-d oral exposure, study II (** Chiang and Flaws 2019 **)**					
*Body and organ weights*	CES	BMDL	BMD	BMDU	BMD/BMDL	BMDU/BMDL
Final body weight, g	−5%	0	241	191	NA	NA
**Body weight change, %**	**+5%**	**191**	**352**	**3,048**	**1.8**	**15.9**
Initial weight, g	NDR					
Ovary weight, g	+5%	0	93	891	NA	NA
Ovary weight, %	+5%	0	88	778	NA	NA
**Uterus weight, g**	**−5%**	**54.5**	**182**	**1,238**	3.3	22.7
**Uterus weight, %**	**−5%**	**60.4**	**190**	**1,418**	3.2	23.5
**Liver, g**	**−5%**	**183**	**218**	**420**	1.2	2.3
**Liver, %**	**−5%**	**188**	**225**	**463**	1.2	2.5

The BMDL, BMD, and BMDU units are mg DEHP/kg bw/d. A positive CES denotes an upward/increasing dose–response, whereas a negative CES a downward/decreasing dose–response relationship. Bolded figures highlight the accepted models according to the employed EFSA QC criteria, and the two lowest BMDLs and BMDs per study are underlined. NDR, no dose–response; NA, not available/not possible to estimate.

### Most female reproductive endpoints show dose-dependent changes

Altogether, the retrieved raw data for 42 endpoints across four individual animal studies (published in six different articles) were subjected to BMD analysis (Tables 3 and 4). For follicle counts, raw counts within categories reported by the authors were considered along with broader summary categories indicating gonadotropin-independent follicles (primordial and primary follicles) and gonadotropin-dependent follicles (preantral and antral). Interestingly, BBMD modeling indicated dose-dependent changes in all but one endpoint; initial body weights in the 10-d oral exposure study II, which as a baseline measure is not expected to show any responses either. Although models could be fitted to all other endpoints, some credible intervals were large, denoting uncertainty. In total, 28 models met the EFSA QC criteria and were included in the final results.

The sensitivities of the different endpoints varied markedly within each study (Tables 3 and 4). In the 10-d oral exposure study I (Hannon et al. 2014), BMDLs ranged from 9 to 142 mg DEHP/kg bw/d, with BMDs ranging from 31 to 240 mg DEHP/kg bw/d. For the 30-d oral exposure study (Hannon et al. 2014), BMDLs ranged from 26 to 176 mg DEHP/kg bw/d, and BMDs ranged from 97 to 211 mg DEHP/kg bw/d. In the 30-d dietary study (Laws et al. 2023; Safar et al. 2023; Santacruz-Márquez et al. 2024), BMDLs ranged from 23 to 246 mg DEHP/kg bw/d with BMDs ranging from 128 to 288 mg DEHP/kg bw/d. Lastly, in the 10-d oral exposure study II (Chiang and Flaws 2019), BMDLs ranged from 55 to 191 mg DEHP/kg bw/d, and BMDs ranged from 182 to 352 mg DEHP/kg bw/d.

### Serum hormone levels and follicle counts are more sensitive endpoints than organ weights and estrous cycle patterns

In general, the serum hormone levels and follicle counts returned the lowest BMDLs and BMDs, while the estrous cyclicity endpoints led to the highest BMDLs and BMDs and the widest credible intervals (Table 3). In the oral exposure studies focusing on HPO endpoints (Hannon et al. 2014), serum progesterone displayed the lowest BMDL values; 9 mg DEHP/kg bw/d for the 10-d study I and 26 mg DEHP/kg bw/d for the 30-d study, although the former with increasing and the latter decreasing dose-dependent changes. In the 30-d dietary exposure study (Laws et al. 2023; Safar et al. 2023; Santacruz-Márquez et al. 2024), the lowest BMDL value was associated with dose-dependent increasing ovarian primordial follicle counts (23 mg DEHP/kg bw/d). In the 10-d oral exposure study II (Chiang and Flaws 2019), which focused on body and organ weight changes, absolute (and relative) uterine weight changes emerged as the most sensitive endpoints. A dose-dependent reduction in uterine weights was observed in response to DEHP exposure, with a BMDL of 55 mg DEHP/kg bw/d.

The direction of the dose–response association, either increasing or decreasing, was not always in agreement across the studies. For serum progesterone levels, there was a dose-dependent increase in the 10-d study I, while it decreased in the 30-d oral and dietary studies (Hannon et al. 2014; Laws et al. 2023; Safar et al. 2023; Santacruz-Márquez et al. 2024). Similarly, for the ovarian primordial follicle counts, there was a DEHP-associated dose-dependent increase in the 30-d dietary exposure study (Laws et al. 2023; Safar et al. 2023; Santacruz-Márquez et al. 2024), while it decreased in the 10- and 30-d oral exposure studies. For all studies analyzed, there was a greater agreement in the direction of the dose-responses for the 10- and 30-d oral exposure studies, in comparison to the same endpoints in the dietary exposure study. The reproductive system is regulated by complex positive and negative feedback loops involving the ovaries, steroid hormones, and estrous cycles, causing the direction of changes to vary depending on the assessment time point. For instance, an early increase in progesterone and estradiol levels (as observed in the 10-d study) likely triggers negative feedback on pituitary gonadotropin secretion, which subsequently reduces follicle growth and lower steroid levels at later time points (as observed in the 30-d study) (Table 3).

Based on the BMDLs and BMDs across all studies, the relative sensitivity of endpoints is as follows: Serum progesterone>follicle counts>uterine weight ≈ serum estradiol>estrous cyclicity. These findings suggest that serum progesterone levels and histological assessment of follicle counts provide valuable data for risk assessment, detecting DEHP-related effects at lower doses than other endpoints.

### Extraction of data from ECHA dossiers

To compare the results of the current study with the existing regulatory risk assessment of DEHP under the European chemicals regulation, REACH, we retrieved data from two public REACH registration dossier databases: The “Registered substances factsheet” and the “ECHA CHEM database.” We also scrutinized the retrieved data to examine the type of female reproductive endpoints that are commonly used, or at a minimum, referenced in regulatory risk assessments. Our database analyses revealed discrepancies between the “Registered substances factsheet” and the newer “ECHA CHEM” database. Specifically, some studies from the discontinued “Registered substances factsheet” were missing in corresponding sections of the “ECHA CHEM” database. Consequently, all repeated dose and reproductive toxicity study datasets found in both databases are listed, with duplicates avoided through careful comparison of study details. The full registration dossier in the “Registered substances factsheet” included 77 repeated dose toxicity (oral route) datasets, categorized as follows: Three “Key studies,” 31 “supporting studies,” and 43 under “other information” (see Fig. 1 and Table S2). For reproductive toxicity, there were 42 studies, with two labeled as “Key studies,” 29 as “supporting studies,” and 11 as “other information” (Fig. 1 and Table S5).

In the “ECHA CHEM” database, the lead registrant’s full registration dossier contained 13 datasets for repeated dose toxicity (oral route), including four “Key studies” and nine “supporting data” (Fig. 1 and Table S3). For reproductive toxicity, the “ECHA CHEM” database contained 14 datasets on reproductive (fertility) toxicity, with one “Key study” and 13 “supporting data” entries (Fig. 1 and Table S6).

After applying the exclusion criteria described in Dossier data section, 13 studies for oral repeated dose toxicity (Table 5 and Table S1) and 13 studies for reproductive toxicity (Table 6 and Table S4) were included. Additionally, Table 7 provides details for the six key datasets, for repeated dose toxicity and reproductive (fertility) toxicity, where no exclusion criteria were applied. Finally, there were only two studies for which NOAEL/LOAEL were derived based on female endpoints. Both these studies were reproductive toxicity studies in which only female animals were used (Table S5). However, the study designs in these were quite unconventional and were therefore excluded (Table S5). In the reproductive toxicity studies analyzed, progesterone levels were measured in only one study, and follicle counts were assessed in just four studies (Table S5). Similarly, estrous cyclicity and ovarian or uterine weights were assessed in 6 and 13 studies, respectively (Table S5). This highlights how rarely female reproductive endpoints are included in reproductive toxicity studies of regulatory relevance.

**Table 5.** A comparison of the repeated dose toxicity NOAELs extracted from the ECHA databases, where DEHP exposure was tested *in vivo* for shorter than 100 d, for studies that included female animals.

Dataset	Species	Duration (d)	NOAEL (mg/kg bw/d)	LOAEL (mg/kg bw/d)
4	Mouse	90	100 mg/kg bw/d	
7	Rat	14		2,000 mg/kg bw/d
11	Rat	16	42 mg/kg bw/d	
17	Rat	14	50 mg/kg bw/d	
20	Rat	21		11 (males) and 12 mg/kg bw/d (females)
22	Rat	90	3.7 (males) and 4.2 mg/kg bw/d (females)	
25	Rat	14	1,500 mg/kg bw/d	
28	Rat	3, 7, 14, 28		50 mg/kg bw/d
30	Monkey	14		2,000 mg/kg bw/d
31	Rat	7, 21		2,500 mg/kg bw/d
32 (Registered substances factsheet) and 13 (ECHA CHEM)	Rat	90	320 mg/kg bw/d	
33	Rat	28		50 mg/kg/d
44	Rat	90		63 (males) and 73 mg/kg/d (females)
57	Rat	3	50 to 80 mg/kg bw/d	
63	Monkey	91	500 mg/kg bw/d	
67	Rat	7, 21		80 mg/kg bw/d
Key dataset 3 (ECHA CHEM)	Rat	90	3.7 mg/kg bw/d	
Key dataset 1 (Registered substances factsheet) and 4 (ECHA CHEM)	Monkey	91	500 mg/kg bw/d	
7 (ECHA CHEM)	Mouse	91	100 mg/kg bw/d	

**Fig. 3.**
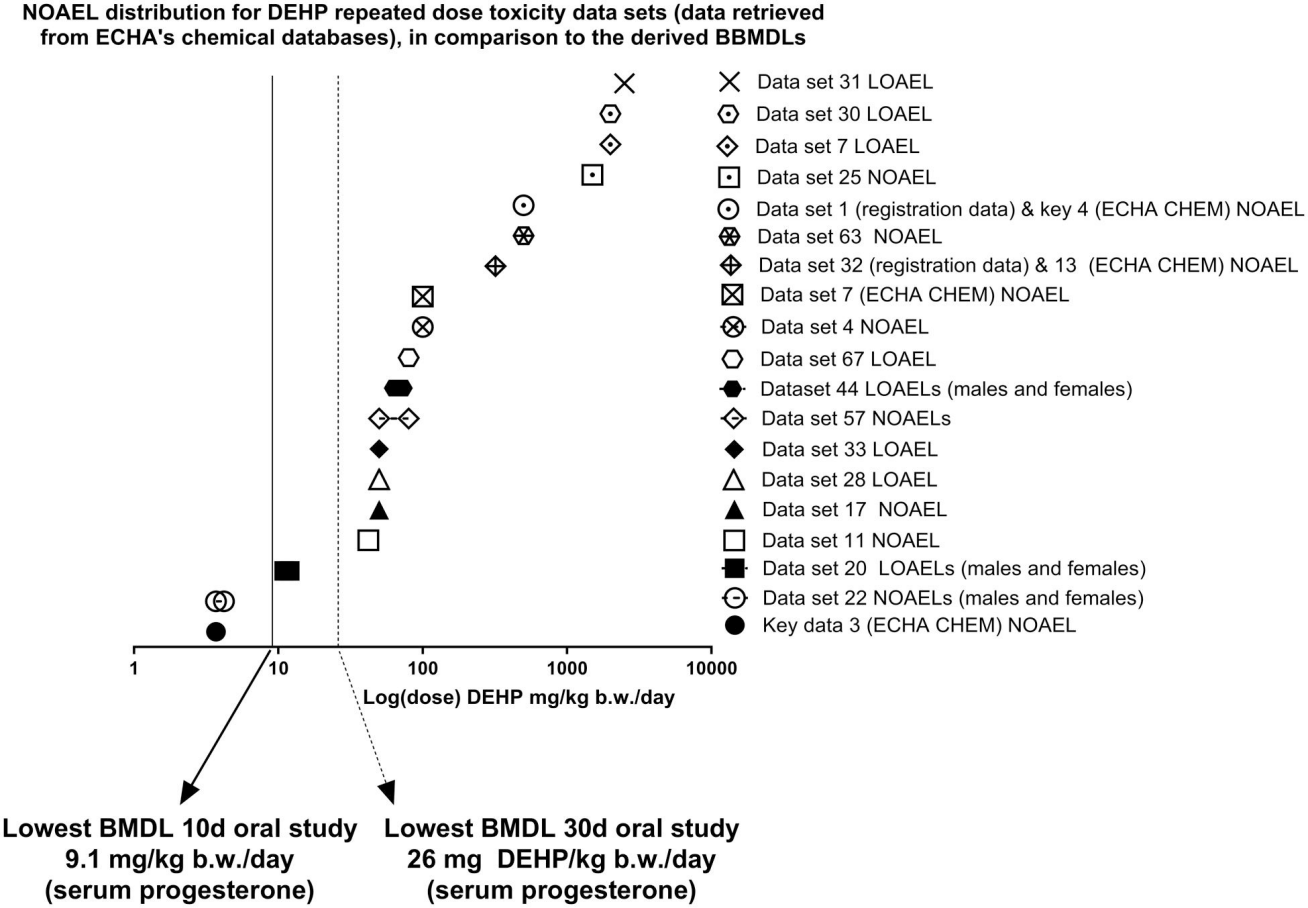
Comparison of the lowest BBMDLs obtained from the 10- and 30-d oral exposure studies, in relation to NOAELs and LOAELs from the extracted repeated dose toxicity studies. The dataset numbering is the same as in Table 5 (and Tables S1 to S3).

The dataset numbers are the same as in Fig. 3, and further details are presented in Table S1.

**Table 6.** A comparison of the reproductive (fertility) toxicity NOAELs extracted from the ECHA databases, where DEHP exposure was tested *in vivo*, for shorter than 100 d, for studies that included female animals.

Dataset	Species	Duration (d)	NOAEL	LOAEL
3	Mouse	98		150 mg/kg bw/d
20 (Registered substances factsheet) and 7 (ECHA CHEM)	Mouse	17	≥95 mg/kg bw/d	
12	Mouse	2, 4, 7, 14, 21, 28, and 30	140 − 493 mg/kg bw/d	
28 (Registered substances factsheet) and 13 ECHA CHEM	Rat	10		1,500 mg/kg bw/d
15	Rat	90	3.7 mg/kg bw/d	
18	Rat	90	1,060 mg/kg bw/d	
19	Mouse	7 + 98	14 mg/kg bw/d	
22	Monkey	91	2,500 mg/kg bw/d	
26	Rat	90	320 mg/kg bw/d	
29 (Registered substances factsheet) and 14 ECHA CHEM	Rat	1 − 12		2,000 mg/kg bw/d
31	Mice	45	55 to 58 mg/kg bw/d	
35	Rat	22 (doses on d1, 5 and 10)		4,900 mg/kg bw/d

**Fig. 4.**
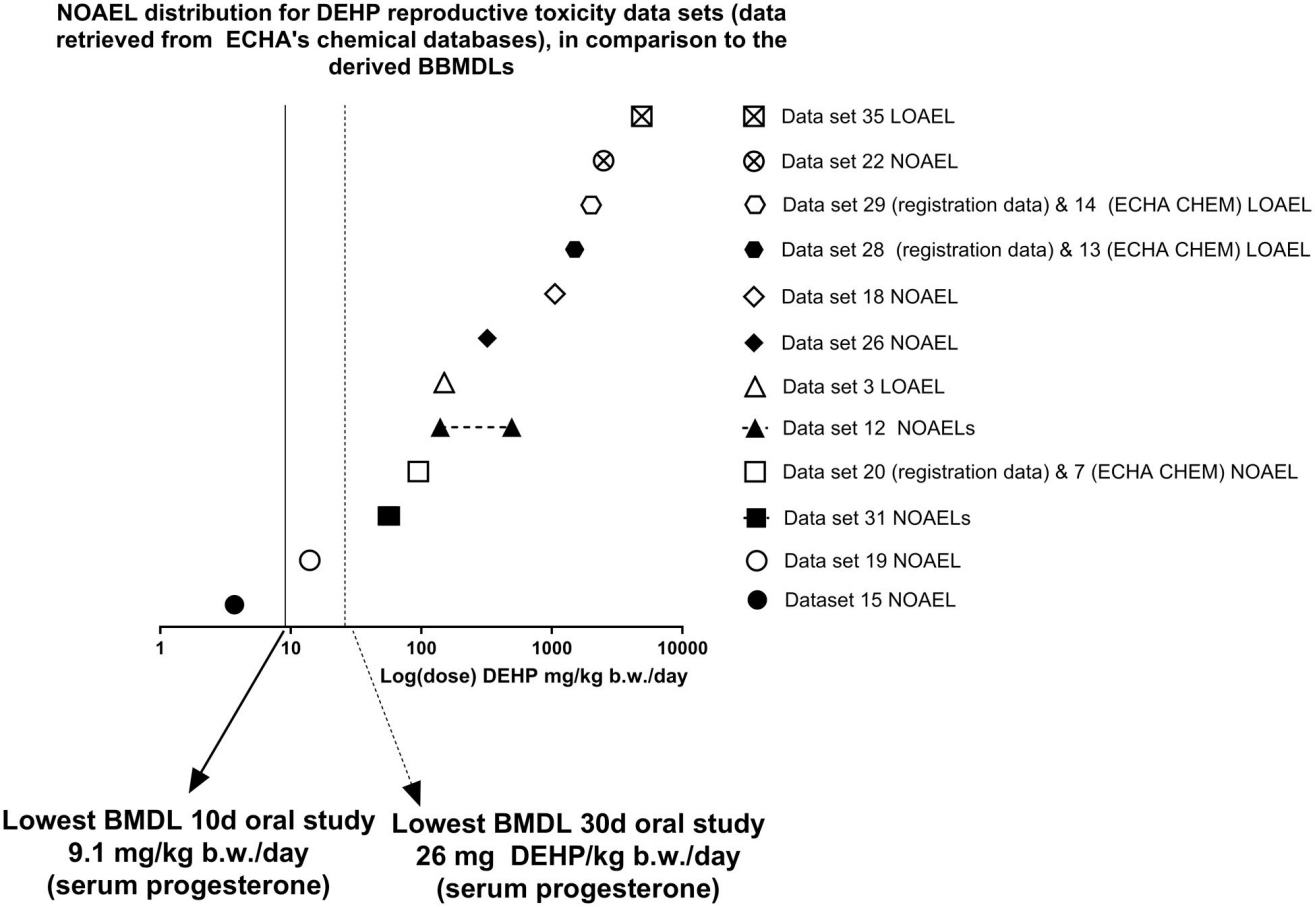
Comparison of the lowest BBMDLs obtained from the 10- and 30-d oral exposure studies, in relation to NOAELs from the extracted reproductive toxicity datasets. The dataset numbering is the same as in Table 6 (and Tables S4 to S6).

The dataset numbers are the same as in Fig. 4 and further details are given in Table S4.

**Table 7.** A comparison of the repeated dose and reproductive toxicity NOAELs extracted from the key studies in ECHA databases and the obtained BMDLs associated with DEHP toxicity *in vivo*.

Repeated dose toxicity			
	DEHP NOAEL (mg/kg bw/d)	Dosing	Findings
**Key data 1 (Registered substances factsheet) and Key data 4 (ECHA CHEM)**	500 mg/kg bw/d	13 wk (91 d), OECD TG 409, oral intake in Marmoset monkeys, m + f, at dose levels 0, 100, 500, and 2,500 mg/kg bw/d.	NOAEL established based on DEHP exposure associated with decreased body weight gain in male and female monkeys.
**Key data 2 (Registered substances factsheet and ECHA CHEM)**	19.2 mg/kg bw/d (males) and 23.8 mg/kg bw/d (females) = 100 ppm	104 wk, OECD TG 453, intake via feed in mice, m + f, at dose levels 0, 100, 500, 1,500, and 6,000 ppm.	NOAEL established based on DEHP exposure-associated liver peroxisome proliferation and increased body weight in male mice.
**Key data 3 (Registered substances factsheet) and key data 1 (ECHA CHEM)**	28.9 mg/kg bw/d (males and 36.1 mg/kg bw/d for females) = 500 ppm	104 wk, OECD TG 453, intake via feed in rats, m + f, at dose levels 0, 100, 500, 2,500, and 12,500 ppm.	NOAEL established based on DEHP exposure-associated liver peroxisome proliferation and increased body weight in male mice.
**Key data 3 (ECHA CHEM)**	3.7 mg/kg bw/d = 50 ppm	13 wk (90 d), OECD TG 408, intake via feed in rats, m + f, at dose levels 0, 5, 50, 500, and 5,000 ppm.	NOAEL established based on DEHP exposure-associated with mild to moderate Sertoli cell vacuolation in the testes.

Reproductive toxicity studies			
	DEHP NOAEL	Dosing	Findings
**Key data 1 and 2 (Registered substances factsheet)**	3 to 5 mg/kg bw/d (F0) = 100 pm	3-generation study in rats, OECD TG 416, intake via feed, m + f Doses: 1.5, 10, 30, 100, 300, 1,000, 7,500, and 10,000 ppm	NOAEL established based on DEHP-exposure associated developmental toxicity effects. Reproductive effects were noted in the 7,500 and 10,000 ppm groups. **Female-specific findings:** Reproductive effects included a decrease in litter size and pup weight, and vaginal opening delay.
**Key data 1 (ECHA CHEM)**	≈ 77 mg/kg bw/d (F0) = 1,000 ppm	3-generation study in rats, OECD TG 416, intake via feed, m + f Doses: 1.5, 10, 30, 100, 300, 1,000, 7,500, and 10,000 ppm	NOAEL established based on DEHP-exposure associated impaired fertility and litter parameters noted (males and females) and above, and decreased various sperm endpoints in male rats, at 7,500 ppm and above. **Female-specific findings**: Reproductive effects included a decrease in pup weight, uterus, cervix, and vagina weights and increased anogenital distance to pup weight in female rats.
	**DEHP NOAEL**	**Dosing**	**Findings**
**This study**	9 to 60 mg/kg bw/d	10 and 30 d, oral and dietary exposure, female mice only. Doses 0.02 to 240 mg/kg bw/d.	**Female-specific findings**: Serum (progesterone and estradiol) hormones, uteri weight, and follicle counts.

#### Registered substances factsheet database

Out of the 77 repeated toxicity studies, 12 were longer than 100 d or had unclear exposure durations and were, therefore, excluded (Fig. 1). Furthermore, 37 studies were excluded as they involved only male animals, and 12 more were excluded for lacking relevant effect levels or failing to report NOAEL/LOAEL values in mg/kg bw/d. Of the remaining 16 studies, 13 were conducted on rats, two on monkeys, and one on mice. Five studies included gross pathology and microscopic examinations of the ovaries and/or the uteri, with one study also reporting weights of the ovaries and uteri. In another study, only uterine weight was assessed. Details of these 16 studies are provided in Table 5, with further information in Table S2.

For the studies on reproduction, there were 42 registered in the substances factsheet database, out of which 8 were longer than 100 d or the exposure time was unclear, and were therefore excluded (Fig. 2). Several studies (19) were excluded as they were conducted only in male animals or had no relevant effect level/did not report NOAEL/LOAEL value in mg/kg bw/d (2). Lastly, one study was excluded as it featured only *in utero*/lactational exposure. The included studies are described in Table 6, with further details in Table S5. Of the 12 final included studies, six examined ovary-related endpoints, primarily weight, and histopathology, while five focused on uterine endpoints.

#### ECHA CHEM database

Out of the 13 repeated toxicity studies in the “ECHA CHEM” database, 4 exceeded 100 d, and 5 involved only male animals (Fig. 1). The remaining studies were conducted on rats, mice, and monkeys. All four repeated toxicity studies performed (histo)pathological examinations on the ovaries and uteri (Table S3). The included studies are summarized in Table 5 and detailed further in Table S3.

For the studies on reproduction on the “ECHA CHEM” database, 5 studies were longer than 100 d, and six were conducted in male animals only, and were therefore excluded (Fig. 2). Out of the three remaining studies, two were conducted on rats and one on monkeys. All of these studies included a histopathological examination of the ovaries or the uteri. The included studies are described in Table 6, and in greater detail in Table S6.

#### Derived no-effect level (DNEL) estimate

A DNEL must be established for all threshold-acting substances registered under the REACH regulation. For DEHP, the DNEL derivation is based on the most sensitive endpoints, namely developmental toxicity of an anti-androgenic nature (small male reproductive organs, minimal testis atrophy, and germ cell depletion) observed in a three-generation oral study in rats (Wolfe and Layton 2003). Based on this study’s findings, ECHA’s restriction report (2016) estimated a DNEL internal dose of 0.034 mg DEHP/kg bw/d departing from a NOAEL of 4.8 mg DEHP/kg bw/d. The total assessment factors were 100 and the correction for absorption was 0.7 (European Chemical Agency (ECHA) 2016). Similarly, EFSA’s Panel on Food Contact Materials, Enzymes and Processing Aids (CEP) estimated a Tolerable Daily Intake value of 0.05 mg DEHP/kg bw/d, based on testicular toxicity and developmental toxicity observed in the same study (EFSA CEP 2019; Wolfe and Layton 2003).

Applying the same total factors and correction for absorption as in ECHA’s calculation to the lowest BBMDLs obtained in our study (9.1 and 19.5 mg DEHP/kg bw/d, the 10-d oral exposure study I) (Hannon et al. 2014) (Table 3), DNELs of 0.064 and 0.14 mg DEHP/kg bw/d can be derived for female reproductive toxicity endpoints (serum progesterone and primary follicle counts, respectively).

### Comparison of the ECHA dossier data and derived Bayesian BMD estimates

**Fig. 5.**
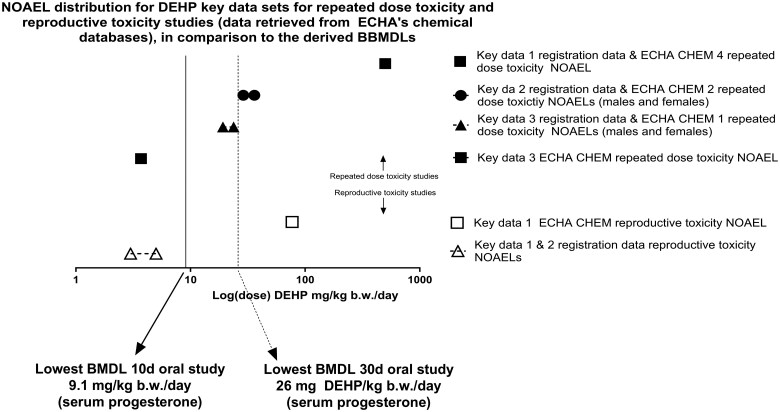
Comparison of the lowest BBMDLs obtained, for the 10- and 30-d oral exposure studies, in relation to NOAELs from the key datasets.

The values extracted from the REACH dossiers were compared with the BMDLs obtained in this study. Overall, the BMDLs derived from the serum progesterone levels, follicle counts, and uterine weight changes were lower than most of the NOAELs and LOAELs extracted from the ECHA databases (Figs 3 to 5, Tables 5 to 7).

For repeated dose toxicity studies, only two out of 19 unique studies (see Table 5) reported a NOAEL lower than the lowest BMDLs obtained via BBMD. Specifically, key dataset 3 (ECHA CHEM) and dataset 22 (Registered substances factsheet database) reported a NOAEL of 3.7 to 4.2 mg DEHP/kg bw/d (males and females) based on testicular effects (Poon et al. 1997) (Fig. 3). In contrast, our study derived BMDLs by BBMD of 9.1 and 26.0 mg DEHP/kg bw/d, for the 10- and 30-d oral exposure studies, respectively (Hannon et al. 2014) (Fig. 3).

For reproductive toxicity studies, only one out of 14 unique studies (see Table 6) reported a NOAEL lower than the lowest BMDLs obtained via BBMD. This was key dataset 15 from the ECHA factsheet database, which reported a NOAEL of 3.7 mg DEHP/kg bw/d based on a 90-d study with histopathological changes in the testes (minimal to mild Sertoli cell vacuolation) (Fig. 4) (Poon et al. 1997). As previously mentioned, BBMD-derived BMDLs were 9.1 and 26.0 mg DEHP/kg bw/d for the 10- and 30-d oral exposure studies, respectively (Hannon et al. 2014).

Finally, comparing this study’s BBMDLs to the NOAELs of the six key datasets from ECHA’s databases, regardless of study length (Table 7), only two cases (one in repeated dose and one in reproductive toxicity studies) reported NOAELs lower than the lowest BBMD L (9.1 mg DEHP/kg bw/d). These were key data 3 (ECHA CHEM) at 3.7 mg DEHP/kg bw/d and key data 1 and 2 at 3 to 5 mg DEHP/kg bw/d (Fig. 5).

## Discussion

### Risk assessment and regulatory relevance of DEHP as a reproductive toxicant

Exposure to environmental contaminants that cause endocrine disruption can lead to adverse effects on fertility, posing a substantial threat to reproductive and overall health (Canipari et al. 2020; Caporossi et al. 2021). In the case of phthalates, such as DEHP, reproductive endpoints are often used for hazard characterization because they are endocrine disruptors (Li et al. 2024).

DEHP’s hazardous profile, including its classification as a reproductive toxicant (Category Repr. 1B) under CLP Regulation ((EC) 1272/2008; European Parliament and Council 2008), GHS hazard statement H360Df (*May damage the unborn child. Suspected of damaging fertility*) and its inclusion in the REACH Annex XIV list as a substance of very high concern and US EPA’s High-Priority Substance underscores its regulatory importance. DEHP has been widely studied, mainly due to its reproductive and developmental toxicity potential. ECHA’s restriction report (2016) DNEL’s internal dose of 0.034 mg DEHP/kg bw/d is in agreement with that of EFSA’s CEP panel (0.05 mg DEHP/kg bw/d), which are similar to the findings of this study (0.064 mg DEHP/kg bw/d), based on antiandrogenic effects (small male reproductive organs, minimal testis atrophy, and germ cell depletion) observed in a developmental study (EFSA CEP 2019; Wolfe and Layton 2003). However, these DNELs rely exclusively on endpoints from the male reproductive system. Significantly lower alternative DNELs of 0.007 and 0.008 mg DEHP/kg bw/d have been proposed by other researchers (Andrade et al. 2006; Christiansen et al. 2010), but these were also based on male reproductive endpoints. Our study shows that, while not lower than the lowest DNELs established on male endpoints, female reproductive endpoints can be similarly or more sensitive than many other reproductive toxicity studies, as illustrated in Figs 4 and 5.

Furthermore, chronic DEHP exposure can cause liver, kidney, and testicular toxicity, as demonstrated in a 2-yr dietary study by David et al. (2000), which reported a NOAEL of 28.9 mg/kg bw/d, which may lead to an even lower DNEL. Altogether, these findings highlight the need for regulatory measures to mitigate DEHP’s potential health risks.

The findings of this study identify serum progesterone levels and ovarian follicle counts as sensitive markers of DEHP exposure, underscoring their relevance in evaluating health risks to female reproductive health. Exposure to DEHP metabolites and other phthalates has been associated with disrupted follicle development, increased follicle loss, and subsequent depletion of the ovarian reserve, which has been linked to female infertility (Panagiotou et al. 2021; Yao et al. 2023). Our analysis of the publicly available REACH registration dossier data showed that these sensitive endpoints are often not included in regulatory risk assessment and they are not an obligatory part of OECD guideline studies either. The inclusion of these female endpoints in DEHP and phthalate risk assessment could provide a clearer picture in comparison to assessments based solely on male (reproductive) endpoints. Moreover, changes in follicle counts and serum progesterone levels are clear indicators of disrupted ovarian homeostasis, which could also have longer-term reproductive health outcomes. However, it is important to keep in mind the possible reversibility and compensation mechanisms associated with progesterone secretion, which is not the case for altered follicle counts. Prioritizing both these endpoints in future assessments and test guidelines could lead to better detection of adverse effects linked to exposure to DEHP, phthalates, and other chemical groups.

When comparing the sensitivity of 10- versus 30-d DEHP exposure, our findings indicate that even short-term exposure can produce significant changes in reproductive toxicity endpoints. Despite identical dosing, the 10-d oral exposure study often resulted in lower BMDs and BMDLs compared with the 30-d study, but also had more excluded results due to increased uncertainty. For estrous cyclicity and ovarian follicle changes, the dose–response direction (upward or downward) was consistent across both durations, whereas serum hormone responses varied. Short-term exposure may reveal more direct targets of DEHP, such as increased progesterone and estradiol levels and stimulated growth activation of gonadotropin-independent follicles. In contrast, longer-term exposure likely involves effects within the entire HPO axis, such as negative pituitary feedback in response to increased progesterone and estradiol levels, which subsequently reduces the growth of gonadotropin-sensitive follicles and lowers estradiol and progesterone levels.

These findings suggest that a standardized 30-d study could capture many of the dose-responses observed in shorter studies but with reduced uncertainty around the effect-inducing doses (reflected in narrower credible intervals). This insight is crucial because traditional assessments often overlook the potential impacts of brief exposures, which may reveal important vulnerabilities in the HPO axis. Understanding these short-term effects helps us detect disruptions in ovarian function and hormonal balance, which might otherwise be hard to detect in longer exposure studies.

Interestingly, when comparing the 30-d oral and the dietary exposure studies, the direction of the dose-responses was the same for estrous cyclicity and serum hormones, but not for ovarian follicles. This discrepancy in ovarian follicle response between exposure routes warrants further investigation. The continuous nature of dietary exposure (animals eat *ad libitum*), differences in the bioavailability and metabolic processing between the two administration routes might explain the differences observed in the outcomes, especially in the case of follicular development. In the 30-d dietary study, all follicular development endpoints revealed a positive dose–response association with DEHP exposure, whereas both the 10- and 30-d oral showed a negative dose–response association. These findings highlight the critical importance of employing multiple exposure paradigms when assessing EDCs, as the method of administration can significantly influence the magnitude and direction of effects on reproductive parameters. On the other hand, internal exposure assessment in these settings could have been helpful, as confirmatory urine analysis of phthalate exposure levels was not performed. Further research is warranted to better understand the mechanistic basis for these divergent responses and to evaluate which exposure route more closely reflects environmentally relevant scenarios for risk assessment purposes. Such studies will enhance our understanding of the compound’s potential impact on reproductive health and support more accurate regulatory decision-making. Lastly, comparing the 10-d oral dosing study II with the other studies reveals that gross changes, such as organ weights, require higher DEHP doses to detect effects compared with functional reproductive toxicity endpoints like estrous cyclicity, serum hormone levels, and ovarian follicle changes. Among organ weights, absolute and relative uterine weight changes were the most sensitive endpoints, reflecting the uterus’ known hormone sensitivity. Interestingly, ovarian weights, a common endpoint in female reproductive toxicity assessments, proved insensitive to DEHP exposure.

The observed hierarchy of sensitivity across various endpoints emphasizes the need for a comprehensive toxicological assessment approach that includes both gross anatomical changes and more subtle physiological alterations. To this day, OECD test guidelines (e.g. TG 408, 416, and 453) (OECD 2018a, 2018b) focus on organ weight changes and other insensitive endpoints (such as estrous cyclicity), and should consider the inclusion of more sensitive markers of reproductive toxicity such as serum hormones and ovarian histology (for example follicle counts). These findings also highlight the potential for DEHP to exert effects on reproductive function at doses lower than those required to induce overt organ weight changes, which has significant implications for risk assessment and the establishment of safety thresholds.

A comparison of this study’s findings and those obtained using a one-way analysis of variance (ANOVA) (Hannon et al. 2014) reveal a stark contrast. BBMD modeling detected a greater number of endpoints and at lower doses, than the traditional ANOVA approach. In particular, BBMD identified dose-dependent changes in estrous cyclicity and folliculogenesis in both the 10- and 30-d studies, capturing these effects more readily than ANOVA. This suggests that ANOVA requires more marked changes to reach statistical significance, as seen with the % estrus and primary follicles changes in the 10- and 30-d studies (Hannon et al. 2014). This discrepancy in sensitivity highlights the limitations of relying solely on traditional statistical methods for (reproductive) toxicological assessments, which are limited to the dose groups chosen before the study start, while BBMD can perform interpolation between dose groups. BMD modeling demonstrates a greater ability to detect subtle, biologically relevant dose–response relationships. The enhanced sensitivity of this method can have significant implications for regulatory decision-making and risk assessment, allowing the identification of potential adverse effects more precisely. By capturing a broader range of endpoints affected by DEHP exposure, it provides a more comprehensive understanding of the compound’s toxicological profile. Adopting these more sensitive analytical tools could lead to more protective regulatory standards and a more nuanced understanding of dose–response relationships in reproductive toxicology. These findings underscore the importance of employing advanced statistical techniques, such as Bayesian approaches, alongside conventional methods in toxicological studies.

Connecting our findings to current DEHP risk assessments reveals a critical gap: Much of the existing research and regulations focus primarily on male reproductive toxicity (US EPA 2000; European Chemicals Agency (ECHA) 2021a). Further studies should consider female reproductive health endpoints because changes can occur and be detected at lower doses than in their male counterparts. This increased sensitivity is important for chemical risk assessment and to public health policy (i.e. to ultimately protect female reproductive health). The observed effects on estrous cyclicity, serum hormone levels, and ovarian folliculogenesis at low doses highlight the regulatory challenge to protect more sensitive populations and the need for a comprehensive approach to DEHP evaluation. To address this challenge, future research could focus on (i) comparative studies assessing male and female reproductive endpoints under identical DEHP exposure conditions, including short and longer-term scenarios; (ii) mechanistic studies to explore the pathways underlying female sensitivity to DEHP; and (iii) development of sex-specific biomarkers for early detection of reproductive toxicity.

Incorporating these endpoints into regulatory assessments would provide a more accurate evaluation of DEHP’s health impacts and contribute to more protective chemical safety regulations for both male and female reproductive health. Additionally, we should consider exploring nonregulatory endpoints that might reveal even more sensitive measures of reproductive toxicity. For instance, examining biomarkers related to oxidative stress, inflammation, and apoptosis in ovarian tissues could provide deeper insights into how DEHP disrupts reproductive health (Fu et al. 2021; Panagiotou et al. 2021). These additional measures, which do not represent a classic endocrine mode of action, could complement the traditional endpoints, giving us a fuller understanding of the impacts of endocrine disruptors and reproductive toxicants like DEHP on female fertility.

In summary, this study calls for a shift in DEHP risk assessment to include female-specific endpoints and consider short-term exposures, which can reflect longer-term exposure scenarios. Refining test guidelines to incorporate more sensitive endpoints, particularly for female reproductive health, can improve the detection of reproductive toxicity, enhance protection of reproductive health, and support the develop more effective regulatory strategies to address risks from DEHP and similar environmental contaminants.

### Bayesian BMD estimates in comparison to the ECHA databases NOAEL estimates

BBMD estimates in this study were compared with the NOAELs and LOAELs in ECHA databases (“Registered substances factsheet” and “ECHA CHEM,” see methods section 3.2 ECHA dossier data extraction results). BBMDLs in this study were generally lower than NOAELs and LOAELs registered in the ECHA databases, especially for the serum progesterone endpoint in both the 10- and 30-d oral exposure studies, indicating it as a highly sensitive marker of DEHP exposure (Tables 5 to 7 and Figs 3 to 5) and that it can reliably detect early effects of reproductive toxicity. BBMD modeling’s ability to interpolate between doses provides additional sensitivity, revealing early reprotoxic effects that NOAEL/LOAEL approaches may overlook. However, it is important to note that ECHA’s public data summaries may not include all available data, presenting a limitation. Serum progesterone and follicle count changes, as analyzed by BBMD, emerge as sensitive markers for DEHP-induced reproductive toxicity, offering early detection of adverse effects. This aligns with prior studies on DEHP’s impact on ovarian function and reproductive health (Hannon et al. 2014; Varik et al. 2024). However, caution should be exercised due to the limited number of studies analyzed (four), and therefore research is needed to reproduce these findings, integrate multiple endpoints, and consider interspecies differences when translating these findings to human risk assessment.

The historical lack of female reproductive toxicity-related endpoints in previous studies can partially be explained by the lack of mention of these endpoints in previous versions of OECD-standardized study guidelines. The previous 1998 version of the OECD Test No. 408 (repeated dose 90-d oral toxicity study in rodents) included only the gross necropsy of the ovaries, and both gross necropsy and histopathological evaluation of the uteri, whereas the revised 2018 version recommends both gross- and histopathological evaluation as well as organ weight measurements of both the ovaries and the uteri (OECD 2018b). The 2008 revised version of the OECD Test No. 407 (repeated dose 28-d oral toxicity study in rodents) recommends both gross- and histopathological examination of both the ovaries and the uterus (OECD 2008). In addition, the current version of this test guideline includes endpoints recommended for the detection of endocrine disrupters, where the histopathological evaluation of the ovaries and the uterus are mandatory endpoints, and ovarian and uterine weights, as well as estrous cyclicity, are mentioned as optional endpoints (TG 407) (OECD 2018a). Similarly, for the OECD TG 408, the same mandatory parameters are included, being estrous cyclicity and circulating levels of reproductive hormones as optional endpoints (OECD 2018b). However, this study’s findings demonstrate that serum progesterone, a normally optional endpoint, and quantitative histopathological evaluation of the ovaries can improve the sensitivity and early detection of reproductive toxicity effects of DEHP and phthalate exposure. Thus, including these endpoints and adopting BBMD as a statistical modeling method could lead to lower PODs, ultimately resulting in more protective regulatory exposure limits and improved public health outcomes.

In conclusion, this study highlights the value of Bayesian BMD modeling over NOAEL-based risk assessment and underscores the sensitivity of serum progesterone levels and follicle counts as markers of DEHP-induced reproductive toxicity. These findings offer a refined understanding of DEHP’s toxicological profile with important implications for more protective regulatory decisions.

### Strengths and limitations

This study offers notable strengths in evaluating DEHP’s effects on female reproductive toxicity through a re-analysis of raw data from published studies and an in-depth analysis of ECHA databases. By analyzing multiple endpoints—estrous cyclicity, serum hormones, ovarian follicle counts, body, and organ weights—it provides a comprehensive view of endpoint sensitivity and DEHP toxicity. The study’s use of environmentally relevant doses—ranging from the U.S. EPA reference dose to estimated occupational human exposure levels—enhances its applicability to real-world scenarios (US EPA 2000). Including both oral administration and dietary exposure routes across different durations (10 and 30 d) allows for nuanced insights into how exposure methods and timeframes may influence toxicity. BBMD modeling strengthens the dose–response assessment by accounting for model uncertainty, and consistent experimental conditions across studies reduce interstudy variability.

Despite these strengths, several limitations should be considered. Studies in the ECHA databases vary in quality and reliability, as not all were conducted for regulatory purposes following standardized test guidelines. Moreover, the described effects were observed in CD-1 mice, and while it can be speculated that similar dose–response relationships could be derived for non-CD-1 mice and other animal models, such data were not modeled in this study. The exclusive use of CD-1 female mice in the studies selected for BMD modeling, may limit the generalizability of findings to other species, including humans and nonrodents. All of the analyzed studies were also conducted in a singular location, which might further limit the generalizability of these findings. Furthermore, the focus on short-term exposure (10 and 30 d) does not address the potential effects of long-term or chronic exposure, which may be more relevant to human exposure patterns. Inter-individual differences might also occur, due to subjectivity in ovarian follicle categorization and estrous cyclicity monitoring. Moreover, the endpoints identified as most sensitive, serum progesterone and follicle counts, are nonapical endpoints, whereas NOAELs tend to be based on apical endpoints (observable biological effects occurring at the whole organism level). Additionally, the study’s focus on adult, fertile, female mice does not account for potential differences in susceptibility at various life stages, particularly during development or reproductive senescence. While the study provides a comprehensive analysis of various endpoints, it does not delve into the underlying molecular mechanisms of DEHP toxicity, indicating a possible knowledge gap. Additionally, the study focuses solely on DEHP exposure, whereas real-world scenarios often involve exposure to complex mixtures of phthalates and other endocrine disruptors, which should be considered. The absence of a recovery period postexposure limits insights into the potential reversibility of observed effects. Lastly, while a wide range of endpoints were examined, there may be other relevant endpoints that were not included in this analysis, potentially introducing bias in endpoint selection.

This study’s strengths significantly enhance its relevance and impact. It offers a comprehensive analysis of multiple reproductive endpoints, modeled human-relevant doses, and exposure routes, and employs BBMD modeling for interpolation and POD derivation. Consistent experimental conditions across studies strengthen result comparability, despite some limitations inherent to animal studies. Overall, the strengths of this study outweigh its limitations, and our results contribute to a deeper understanding of DEHP’s reproductive toxicity and pave the way for more informed regulatory decisions regarding female reproductive health.

### Future perspectives

This study’s findings underscore the need for a paradigm shift in female reproductive toxicity assessment. Firstly, it shows that the endpoints included in dossiers and/or guideline studies are not the most sensitive. Including more refined measures, like serum progesterone and quantitative histopathological assessment of ovaries including follicle counts, will help in the identification of reproductive toxicants. Secondly, it shows the relevance of performing shorter-term studies, potentially with the same number of animals divided into more dose groups, that potentially reflect the findings of longer-term studies (with fewer dose groups) (Ringblom et al. 2018). Thirdly, future research could focus on elucidating the molecular mechanisms underlying the differences observed in relation to female sensitivity to DEHP exposure. Longitudinal studies examining the long-term consequences of low-dose DEHP exposure on female fertility, ovarian function, and reproductive lifespan are crucial. Additionally, investigating potential transgenerational effects and epigenetic modifications resulting from DEHP exposure could provide valuable insights into elucidating its potential broader impact on population health. Furthermore, expanding this research to other phthalates, endocrine-disrupting chemicals, and reproductive toxicants would contribute to a more comprehensive understanding of environmental impacts on female reproductive health. Altogether, these efforts would contribute to the improvement of test guidelines and regulatory frameworks to ensure adequate protection for both male and female reproductive health, following DEHP and phthalate exposure.

## Conclusions

In conclusion, this study demonstrates that short-term exposure studies can reveal early effects consistent with those observed in longer-term studies, and that female reproductive endpoints, particularly follicle counts and serum hormone levels, show greater sensitivity to DEHP exposure than traditional toxicological markers. Notably, the identified sensitive endpoints—progesterone and follicle counts—were recently found to be altered in women with higher DEHP exposure levels (Varik et al. 2024), suggesting their human relevance. Collectively, our findings challenge the current male-centric approach in DEHP risk assessment and underscore the urgent need to integrate female-specific endpoints into regulatory frameworks. Prioritizing these sensitive indicators will improve our ability to detect and address the reproductive risks of DEHP exposure, ultimately helping to protect female reproductive health in an increasingly chemical-laden environment.

## Supplementary Material

kfaf052_Supplementary_Data

## References

[kfaf052-B1] Andrade AJM , Grande SW, Talsness CE, Gericke C, Grote K, Golombiewski A, Sterner-Kock A, Chahoud I. 2006. A dose-response study following *in utero* and lactational exposure to di(2-ethylhexyl) phthalate (DEHP): effects on rat brain development. Toxicology. 228:85–97. 10.1016/j.tox.2006.08.006 16996189

[kfaf052-B2] Bellavia A , Zou R, Björvang RD, Roos K, Sjunnesson Y, Hallberg I, Holte J, Pikki A, Portengen L, Koekkoek J, et al 2023. Association between chemical mixtures and female fertility in women undergoing assisted reproduction in Sweden and Estonia. Environ Res. 216:114447. 10.1016/j.envres.2022.114447 36181890 PMC9729501

[kfaf052-B3] Canipari R , De Santis L, Cecconi S. 2020. Female fertility and environmental pollution. Int J Environ Res Public Health. 17:8802. 10.3390/ijerph17238802 33256215 PMC7730072

[kfaf052-B4] Caporossi L , Vigano P, Paci E, Capanna S, Alteri A, Campo G, Pigini D, De Rosa M, Tranfo G, Papaleo B. 2021. Female reproductive health and exposure to phthalates and bisphenol A: a cross sectional study. Toxics. 9:299. 10.3390/toxics9110299 34822691 PMC8622554

[kfaf052-B5] Chiang C , Flaws JA. 2019. Subchronic exposure to di(2-ethylhexyl) phthalate and diisononyl phthalate during adulthood has immediate and long-term reproductive consequences in female mice. Toxicol Sci. 168:620–631. 10.1093/toxsci/kfz013 30649530 PMC6432868

[kfaf052-B6] Chiang C , Lewis LR, Borkowski G, Flaws JA. 2020a. Late-life consequences of short-term exposure to di(2-ethylhexyl) phthalate and diisononyl phthalate during adulthood in female mice. Reprod Toxicol. 93:28–42. 10.1016/j.reprotox.2019.12.006 31904422 PMC7138709

[kfaf052-B7] Chiang C , Lewis LR, Borkowski G, Flaws JA. 2020b. Exposure to di(2-ethylhexyl) phthalate and diisononyl phthalate during adulthood disrupts hormones and ovarian folliculogenesis throughout the prime reproductive life of the mouse. Toxicol Appl Pharmacol. 393:114952. 10.1016/j.taap.2020.114952 32165126 PMC7138141

[kfaf052-B9] Christiansen S , Boberg J, Axelstad M, Dalgaard M, Vinggaard AM, Metzdorff SB, Hass U. 2010. Low-dose perinatal exposure to di(2-ethylhexyl) phthalate induces anti-androgenic effects in male rats. Reprod Toxicol. 30:313–321. 10.1016/j.reprotox.2010.04.005 20420898

[kfaf052-B11] David RM , Moore MR, Finney DC, Guest D. 2000. Chronic toxicity of di(2-ethylhexyl)phthalate in rats. Toxicol Sci. 55:433–443. 10.1093/toxsci/55.2.433 10828276

[kfaf052-B13] Doull J , Cattley R, Elcombe C, Lake BG, Swenberg J, Wilkinson C, Williams G, van Gemert M. 1999. A cancer risk assessment of di(2-ethylhexyl)phthalate: application of the new U.S. EPA risk assessment guidelines. Regul Toxicol Pharmacol. 29:327–357. 10.1006/rtph.1999.1296 10388618

[kfaf052-B14] Du YY , Fang YL, Wang YX, Zeng Q, Guo N, Zhao H, Li YF. 2016. Follicular fluid and urinary concentrations of phthalate metabolites among infertile women and associations with in vitro fertilization parameters. Reprod Toxicol. 61:142–150. 10.1016/j.reprotox.2016.04.005 27067915

[kfaf052-B34485700] European Chemical Agency (ECHA). 2016. Practical Guide: How to use alternatives to animal testing to fulfil the information requirements for REACH registration. ECHA-16-B-25-EN. https://echa.europa.eu/documents/10162/17250/practical_guide_how_to_use_alternatives_en.pdf/148b30c7-c186-463c-a898-522a888a4404

[kfaf052-B15] European Chemical Agency (ECHA) and European Food Safety Authority (EFSA) with the Technical Support of the Joint Research Centre (JRC); Andersson N, Arena M, Auteri D, Barmaz S, Grignard E, Kienzler A, Lepper P, Lostia AM, Munn S, Parra Morte JM, et al 2018. Guidance for the identification of endocrine disruptors in the context of regulations (EU) No 528/2012 and (EC) No 1107/2009. Efsa J. 16:e05311. 10.2903/j.efsa.2018.5311 32625944 PMC7009395

[kfaf052-B16] European Chemicals Agency (ECHA). 2021a. Annex XV restriction report—four phthalates (DEHP, BBP, DBP, DIBP). [accessed 2024 Aug 9]. https://echa.europa.eu/documents/10162/2700f4f2-579a-1fbe-2c23-311706a3e958

[kfaf052-B17] European Chemicals Agency (ECHA). 2021b. Guidance on registration (version 4.0). ECHA-21-G-05-EN. https://echa.europa.eu/documents/10162/2324906/registration_en.pdf

[kfaf052-B18] European Food Safety Authority Panel on Food Contact Materials, Enzymes and Processing Aids, EFSA CEP. 2019. Update of the risk assessment of di‐butylphthalate (DBP), butyl‐benzyl‐phthalate (BBP), bis (2‐ethylhexyl) phthalate (DEHP), di‐isononylphthalate (DINP) and di‐isodecylphthalate (DIDP) for use in food contact materials. Efsa J. 17:e05838. http://onlinelibrary.wiley.com/doi/10.2903/sp.efsa.2019.EN-1747/full 32626195 10.2903/j.efsa.2019.5838 PMC7008866

[kfaf052-B19] European Food Safety Authority, EFSA. 2022. Guidance on the use of the benchmark dose approach in risk assessment. Efsa J. 20:e07584. 10.2903/j.efsa.2022.7584 36304832 PMC9593753

[kfaf052-B20] European Parliament and Council. 2008. Regulation (EC) No 1272/2008 of the European parliament and of the council of 16 December 2008 on classification, labelling and packaging of substances and mixtures, amending and repealing directives 67/548/EEC and 1999/45/EC, and amending regulation (EC) No 1907/2006. Off J Eur Union. L353:1–1355. http://data.europa.eu/eli/reg/2008/1272/2023-07-31

[kfaf052-B21] Fu X , Han H, Li Y, Xu B, Dai W, Zhang Y, Zhou F, Ma H, Pei X. 2021. Di‐(2‐ethylhexyl) phthalate exposure induces female reproductive toxicity and alters the intestinal microbiota community structure and fecal metabolite profile in mice. Environ Toxicol. 36:1226–1242. 10.1002/tox.23121 33665894 PMC8251547

[kfaf052-B22] Goldman JM , Murr AS, Cooper RL. 2007. The rodent estrous cycle: characterization of vaginal cytology and its utility in toxicological studies. Birth Defects Res B Dev Reprod Toxicol. 80:84–97. 10.1002/bdrb.20106 17342777

[kfaf052-B23] Gupta RK , Singh JM, Leslie TC, Meachum S, Flaws JA, Yao H. 2010. Di-(2-ethylhexyl) phthalate and mono-(2-ethylhexyl) phthalate inhibit growth and reduce estradiol levels of antral follicles in vitro. Toxicol Appl Pharmacol. 242:224–230. 10.1016/j.taap.2009.10.011 19874833 PMC2789888

[kfaf052-B24] Hannon PR , Flaws JA. 2015. The effects of phthalates on the ovary. Front Endocrinol (Lausanne). 6:8–19. 10.3389/fendo.2015.00008 25699018 PMC4313599

[kfaf052-B25] Hannon PR , Niermann S, Flaws JA. 2016. Acute exposure to di(2-ethylhexyl) phthalate in adulthood causes adverse reproductive outcomes later in life and accelerates reproductive aging in female mice. Toxicol Sci. 150:97–108. 10.1093/toxsci/kfv317 26678702 PMC5009616

[kfaf052-B26] Hannon PR , Peretz J, Flaws JA. 2014. Daily exposure to di(2-ethylhexyl) phthalate alters estrous cyclicity and accelerates primordial follicle recruitment potentially via dysregulation of the phosphatidylinositol 3-kinase signaling pathway in adult mice. Biol Reprod. 90:136. 10.1095/biolreprod.114.119032 24804967 PMC4435463

[kfaf052-B27] Hines CJ , Nilsen Hopf NB, Deddens JA, Calafat AM, Silva MJ, Grote AA, Sammons DL. 2009. Urinary phthalate metabolite concentrations among workers in selected industries: a pilot biomonitoring study. Ann Occup Hyg. 53:1–17. 10.1093/annhyg/men066 18948546

[kfaf052-B28] Kavlock R , Boekelheide K, Chapin R, Cunningham M, Faustman E, Foster P, Golub M, Henderson R, Hinberg I, Little R, et al 2002. NTP center for the evaluation of risks to human reproduction: phthalates expert panel report on the reproductive and developmental toxicity of di(2-ethylhexyl)phthalate. Reprod Toxicol. 16:529–653. 10.1016/S0890-6238(02)00032-1 12406494

[kfaf052-B29] Kay VR , Chambers C, Foster WG. 2013. Reproductive and developmental effects of phthalate diesters in females. Crit Rev Toxicol. 43:200–219. 10.3109/10408444.2013.766149 23405971 PMC3604737

[kfaf052-B30] Krotz SP , Carson SA, Tomey C, Buster JE. 2012. Phthalates and bisphenol do not accumulate in human follicular fluid. J Assist Reprod Genet. 29:773–777. 10.1007/s10815-012-9775-1 22538552 PMC3430784

[kfaf052-B31] Laws MJ , Meling DD, Deviney ARK, Santacruz-Márquez R, Flaws JA. 2023. Long-term exposure to di(2-ethylhexyl) phthalate, diisononyl phthalate, and a mixture of phthalates alters estrous cyclicity and/or impairs gestational index and birth rate in mice. Toxicol Sci. 193:48–61. 10.1093/toxsci/kfad030. 36929940 PMC10176245

[kfaf052-B32] Li K , Zhang Y, Li L, Cui K, Li Y, Li C, Dai Y, Xiao W, Wang Q. 2024. Identification of sensitive endpoints for the assessment of phthalates-induced reproductive and developmental toxicity: a literature mining study. Food Chem Toxicol. 188:114686. 10.1016/j.fct.2024.114686 38663762

[kfaf052-B33] Lovekamp TN , Davis BJ. 2001. Mono-(2-ethylhexyl) phthalate suppresses aromatase transcript levels and estradiol production in cultured rat granulosa cells. Toxicol Appl Pharmacol. 172:217–224. 10.1006/taap.2001.9156 11312650

[kfaf052-B34] Messerlian C , Souter I, Gaskins AJ, Williams PL, Ford JB, Chiu YH, Calafat AM, Hauser R, Earth Study Team. 2016. Urinary phthalate metabolites and ovarian reserve among women seeking infertility care. Hum Reprod. 31:75–83. 10.1093/humrep/dev292 26573529 PMC4677966

[kfaf052-B35] Moyer B , Hixon ML. 2012. Reproductive effects in F1 adult females exposed *in utero* to moderate to high doses of mono-2-ethylhexylphthalate (MEHP). Reprod Toxicol. 34:43–50. https://doi.org/10.1016%2Fj.reprotox.2012.02.006 22401849 10.1016/j.reprotox.2012.02.006 PMC3367132

[kfaf052-B36] OECD, Organisation for Economic Co-operation and Development. 2008. Test No. 407: repeated dose 28-day oral toxicity study in rodents, OECD guidelines for the testing of chemicals, Section 4. Paris: OECD Publishing. 10.1787/9789264070684-en

[kfaf052-B37] OECD, Organisation for Economic Co-operation and Development. 2018a. Revised guidance document 150 on standardised test guidelines for evaluating chemicals for endocrine disruption. In OECD series on testing and assessment. Paris, France: Organisation for Economic Co-operation and Development (OECD) Publishing. 10.1787/9789264304741-en

[kfaf052-B38] OECD, Organisation for Economic Co-operation and Development. 2018b. Test No. 408: repeated dose 90-day oral toxicity study in rodents, OECD guidelines for the testing of chemicals, Section 4. Paris, France: OECD Publishing. 10.1787/9789264070707-en

[kfaf052-B39] Panagiotou EM , Ojasalo V, Damdimopoulou P. 2021. Phthalates, ovarian function and fertility in adulthood. Best Pract Res Clin Endocrinol Metab. 35:101552. 10.1016/j.beem.2021.101552 34238683

[kfaf052-B40] Piersma AH , Hakkert B, Muller JJA. 2007. Current innovations in regulatory reproductive toxicity assessment of chemicals. RIVM Report 340700002/2007.

[kfaf052-B41] Poon R , Lecavalier P, Mueller R, Valli VE, Procter BB, Chu I. 1997. Subchronic oral toxicity of di-n-octyl phthalate and di (2-ethylhexyl) phthalate in the rat. Food Chem Toxicol. 35:225–239. 10.1016/S0278-6915(96)00064-6 9146736

[kfaf052-B42] Reed BG , Carr BR. 2000. The normal menstrual cycle and the control of ovulation. In: Feingold KR, Anawalt B, Blackman MR, Boyce A, Chrousos G, Corpas E, de Herder WW, Dhatariya K, Dungan K, Hofland J, et al, editors. Endotext. South Dartmouth (MA): MDText.com, Inc. .

[kfaf052-B43] Ringblom J , Kalantari F, Johanson G, Öberg M. 2018. Influence of distribution of animals between dose groups on estimated benchmark dose and animal welfare for continuous effects. Risk Anal. 38:1143–1153. 10.1111/risa.12929 29084354

[kfaf052-B44] Safar AM , Santacruz-Márquez R, Laws MJ, Meling DD, Liu Z, Kumar TR, Nowak RA, Raetzman RT, Flaws JA. 2023. Dietary exposure to an environmentally relevant phthalate mixture alters follicle dynamics, hormone levels, ovarian gene expression, and pituitary gene expression in female mice. Reprod Toxicol. 122:108489. 10.1016/j.reprotox.2023.108489. 37839492 PMC10873030

[kfaf052-B45] Santacruz-Márquez R , Safar AM, Laws MJ, Meling DD, Liu Z, Kumar TR, Nowak RA, Raetzman LT, Flaws JA. 2024. The effects of short-term and long-term phthalate exposures on ovarian follicle growth dynamics and hormone levels in female micedagger. Biol Reprod. 110:198–210. 10.1093/biolre/ioad137 37812459 PMC10790346

[kfaf052-B46] Shao K , Shapiro AJ. 2018. A web-based system for Bayesian benchmark dose estimation. Environ Health Perspect. 126:017002. 10.1289/EHP1289 29329100 PMC6014690

[kfaf052-B47] Silva ABP , Carreiro F, Ramos F, Sanches-Silva A. 2023. The role of endocrine disruptors in female infertility. Mol Biol Rep. 50:7069–7088. 10.1007/s11033-023-08583-2 37402067 PMC10374778

[kfaf052-B49] Tarvainen I , Soto DA, Laws MJ, Björvang RD, Damdimopoulos A, Roos K, Li T, Kramer S, Li Z, Lavogina D, et al 2023. Identification of phthalate mixture exposure targets in the human and mouse ovary in vitro. Reprod Toxicol. 119:108393. 10.1016/j.reprotox.2023.108393 37160244

[kfaf052-B50] U.S. Environmental Protection Agency (EPA). 1996. Guidelines for reproductive toxicity risk assessment. Federal Register. 61:56274–56322. https://www.epa.gov/sites/default/files/2014-11/documents/guidelines_repro_toxicity.pdf [acessed 22/8/2024].

[kfaf052-B51] U.S. Environmental Protection Agency (EPA). 2000. Toxicological review of di(2-ethylhexyl) phthalate (DEHP) (CAS No. 117-81-7) in support of summary information on the integrated risk information system (IRIS). Washington (DC): U.S. Environmental Protection Agency. [accessed 2024 Aug 11]. https://iris.epa.gov/static/pdfs/0014_summary.pdf

[kfaf052-B52] Varik I , Zou R, Bellavia A, Rosenberg K, Sjunnesson Y, Hallberg I, Holte J, Lenters V, Van Duursen M, Pedersen M, et al 2024. Reduced ovarian cholesterol and steroid biosynthesis along with increased inflammation are associated with high DEHP metabolite levels in human ovarian follicular fluids. Environ Int. 191:108960. 10.1016/j.envint.2024.108960 39173238

[kfaf052-B54] Wolfe GW , Layton KA. 2003. Multi-generation reproduction toxicity study in rats. Unaudited draft: diethylhexylphthalate: multigenerational reproductive assessment when administered to Sprague-Dawley rats in the diet. Gaithersburg (MD): The Immune Research Corporation, TRC Study No. 7244–200.

[kfaf052-B55] Yao Y , Du Y, Guo N, Liu F, Deng T, Li Y. 2023. Associations between urinary phthalate concentrations and antral follicle count among women undergoing in vitro fertilization. Front Endocrinol (Lausanne). 14:1286391. 10.3389/fendo.2023.1286391 38260134 PMC10801055

[kfaf052-B56] Zhou C , Flaws JA. 2017. Effects of an environmentally relevant phthalate mixture on cultured mouse antral follicles. Toxicol Sci. 156:217–229. 10.1093/toxsci/kfw245 28013214 PMC6075604

[kfaf052-B57] Zhou C , Gao L, Flaws JA. 2017. Prenatal exposure to an environmentally relevant phthalate mixture disrupts reproduction in F1 female mice. Toxicol Appl Pharmacol. 318:49–57. 10.1016/j.taap.2017.01.010 28126412 PMC5303666

