# Supplementary material for: Bayesian benchmark dose modeling analysis and derivation of points of departure for female reproductive toxicity following exposure to di(2-ethylhexyl) phthalate (DEHP) — effects on reproductive hormones, folliculogenesis and estrous cyclicity
Source: Toxicol Sci. 2025 Apr 21;206(1):30–45. doi: 10.1093/toxsci/kfaf052 (PMC12198667; doi:10.1093/toxsci/kfaf052)
Supplement: kfaf052_Supplementary_Data [file kfaf052_supplementary_data.zip › Supplementary material.pdf]

Table S1. All included studies from both ECHA's registered factsheet and CHEM databases where repeated dose toxicity of DEHP was investigated *in vivo*. Unless indicated otherwise with "ECHA CHEM", most included studies were retrieved from ECHA registered factsheet database.

Data set	Species	Duration (days)	ovar*	follic*	uter*	proges*	(o)estrou*	NOAEL	LOAEL	NOAEL/LOAEL finding	Exclusion criteria
4 Supporting	Mouse	90	Histopathology	n/a	Histopathology	n/a	n/a	800ppm (100 mg/kg bw)	n/a	Body weight gain ↓	n/a
7 Supporting	rat	14	n/a	n/a	n/a	n/a	n/a	n/a	2 000 mg/kg bw/day	Body weight gain ↓ (males), Kidney weight ↑ (females)	n/a
11 Supporting	rat	16	n/a	n/a	Organ weight	n/a	n/a	42 mg/kg bw/day (male)	n/a	Liver weight ↑	n/a
17 Supporting	rat	14	n/a	n/a	n/a	n/a	n/a	50 mg/kg bw/day	n/a	Hepatocellular cytomegaly	n/a
20 Supporting	rat	21	n/a	n/a	n/a	n/a	n/a	n/a	0,01 %(11 mg/kg bw/day males and 12 mg/kg bw/day females)	Serum triglyceride levels ↑	n/a
22 Supporting	rat	90	Histopathology	no	Histopathology	no	n/a	500 ppm (3.7 mg/kg bw/day males and 4.2 mg/kg bw/day females)	n/a	Testicular toxicity, liver weight ↑	n/a
25 Supporting	rat	14	n/a	n/a	n/a	n/a	n/a	1 500 mg/kg bw/day	n/a	n/a	n/a
28 Supporting	rat	3, 7, 14, 28	n/a	n/a	n/a	n/a	n/a	n/a	50 mg/kg bw/day	Number of peroxisomes ↑	n/a
30 Supporting	monkey	14	n/a	n/a	n/a	n/a	n/a	n/a	2 000 mg/kg bw/day	Relative kidney weights ↓ in females, hepatic catalase activity ↑ in males.	n/a
31 Supporting	rat	7, 21	n/a	n/a	n/a	n/a	n/a	n/a	2 500 mg/kg bw/day	n/a	n/a
32 Supporting	rat	90	Histopathology	n/a	Histopathology	n/a	n/a	6 300 ppm (320 mg/kg bw/day)	n/a	Testes atrophy	n/a
33 Supporting	rat	28	n/a	n/a	n/a	n/a	n/a	n/a	50 mg/kg bw/day (males)	Liver weight ↑ and enzyme activity	n/a
44 Other	rat	90	n/a	n/a	n/a	n/a	n/a	n/a	1 000 ppm (63 mg/kg bw/day males and 73 mg/kg bw/d females)	Liver weight ↑	n/a
57 Other	rat	3	n/a	n/a	n/a	n/a	n/a	50 - 80 mg/kg bw/day	n/a	Liver and kidney weight ↑, body weight ↓	n/a
63 Other	monkey	90	Pathology	n/a	Pathology	n/a	n/a	500 mg/kg bw/day	n/a	Body weight gain ↓	n/a
67 Other	rat	7, 21	n/a	n/a	n/a	n/a	n/a	n/a	0.1% (80 mg/kg bw/day)	Liver weight ↑	n/a
Key 3 (ECHA CHEM)	Rat	90	Gross- and histopathology	n/a	Gross- and histopathology	n/a	n/a	500 ppm (3.7 mg/kg bw/day)	n/a	Testes: mild to moderate Sertoli cell vacuolation from 500 ppm (7/10)	n/a
Key 4 (ECHA CHEM)	Monkey	91	Pathology	n/a	Pathology	n/a	n/a	500 mg/kg bw/day	n/a	Decreased body weight gain at 2 500 mg/kg bw/day	n/a
7 Supporting (ECHA CHEM)	Mouse	90	Pathology	n/a	Pathology	n/a	n/a	800 ppm (100 mg/kg bw/day)	n/a	↓bwg from 3 100 ppm (males) and from 1 600 ppm (females)	n/a
13 Supporting (ECHA CHEM)	Rat	90	Pathology	n/a	Pathology	n/a	n/a	6 300 ppm (320 mg/kg bw/day)	n/a	↓bwg at 25 000 ppm. Testes: atrophy from 12 500 ppm	n/a

Table S2. All identified studies in ECHA's registered factsheet database where repeated dose toxicity of DEHP was investigated *in vivo*. Red font denotes excluded studies, according to the exclusion criteria in the last column. Black font denotes included studies (also listed in Table S1).

Data set	Species	Duration (days)	ovar*	follic*	uter*	proges*	(o)estrou*	NOAEL	LOAEL	NOAEL/LOAEL finding	Exclusion criteria
1 Key	monkey	455	Microscopical examination	n/a	Microscopical examination	n/a	n/a	2 500 mg/kg bw/day	n/a	n/a	Duration
2 Key	mouse	728	Histopathology	n/a	Histopathology	n/a	n/a	100 ppm (19.2 mg/kg bw/day males and 23.8 mg/kg bw/day females)	n/a	n/a	Duration
3 Key	rat	728	n/a	n/a	Weight and gross pathology	n/a	n/a	500 ppm (28.9 mg/kg bw/day males and 36.1 mg/kg bw/day females)	n/a	n/a	Duration
**4 Supporting**	**Mouse**	**90**	**Histopathology**	**n/a**	**Histopathology**	**n/a**	**n/a**	**800 ppm (100 mg/kg bw/day)**	n/a	**Body weight gain ↓**	**n/a**
5 Supporting	rat	63	n/a	n/a	n/a	n/a	n/a	1 000 mg/kg bw/day	n/a	n/a	Males only
6 Supporting	rat	28	n/a	n/a	n/a	n/a	n/a	5 mg/kg bw/day	n/a	Peroxisome proliferation	Males only
**7 Supporting**	**rat**	**14**	**n/a**	**n/a**	**n/a**	**n/a**	**n/a**	**n/a**	**2 000 mg/kg bw/day**	**Body weight gain ↓ (males), Kidney weight ↑ (females)**	**n/a**
8 Supporting	rat	21	n/a	n/a	n/a	n/a	n/a	11 mg/kg bw/day	n/a	Palmitoyl Co A activity ↑	Males only
9 Supporting	rat	21	n/a	n/a	n/a	n/a	n/a	n/a	2%	Body weight ↓, liver weight ↑	Males only
10 Supporting	rat	714	n/a	n/a	n/a	n/a	n/a	n/a	7 mg/kg bw/day	Testes atrophy and inhibition of spermatogenesis	Duration and males only
**11 Supporting**	**rat**	**16**	**n/a**	**n/a**	**Organ weight**	**n/a**	**n/a**	**42 mg/kg bw/day (male)**	**n/a**	**Liver weight ↑**	**n/a**
12 Supporting	mouse	56	n/a	n/a	n/a	n/a	n/a	n/a	1 000 mg/kg bw/day	Thymidine kinase activity ↓	Males only
13 Supporting	rat	3	n/a	n/a	n/a	n/a	n/a	n/a	25 mg/kg bw/day	↑p.enz.act.	Males only
14 Supporting	hamster	14	n/a	n/a	n/a	n/a	n/a	250 mg/kg bw/day	n/a	Liver weight and activities of PCoA and CAT	Males only
15 Supporting	rat	728	n/a	n/a	n/a	n/a	n/a	n/a	6 000 ppm	n/a	Duration
16 Supporting	rat	90	n/a	n/a	n/a	n/a	n/a	n/a	n/a	n/a	No effect level
**17 Supporting**	**rat**	**14**	**n/a**	**n/a**	**n/a**	**n/a**	**n/a**	**50 mg/kg bw/day**	**n/a**	**Hepatocellular cytomegaly**	**n/a**
18 Supporting	rat	n/a	n/a	n/a	n/a	n/a	n/a	n/a	12 000 ppm	Several	Males only
19 Supporting	rat	28	n/a	n/a	n/a	n/a	n/a	n/a	0.02% in the diet (24 mg/kg bw/day).	Liver weight ↑	Males only
**20 Supporting**	**rat**	**21**	**n/a**	**n/a**	**n/a**	**n/a**	**n/a**	**n/a**	**0,01 %(11 mg/kg /day males and 12 mg/kg bw/day females)**	**Serum triglyceride levels ↑**	**n/a**
21 Supporting	rat	28	n/a	n/a	n/a	n/a	n/a	1 000 mg/kg bw/day	5 000 mg/kg bw/day	Testes weight ↓	Males only
**22 Supporting**	**rat**	**90**	**Histopathology**	**no**	**Histopathology**	**no**	**n/a**	**500 ppm (3.7 mg/kg bw/day males and 4.2 mg/kg bw/day females)**	**n/a**	**Testicular toxicity, liver weight ↑**	**n/a**
23 Supporting	rat	119	n/a	n/a	Grossa pathology	n/a	n/a	n/a	0.2% in the diet (143 mg/kg bw/day (males) and 145 mg/kg bw/day (females).	Liver weight ↑	Duration

24 Supporting	mouse	168	n/a	n/a	n/a	n/a	n/a		12 000 ppm	mortality, body weight, relative organ weight, peroxisome proliferation	Duration and males only
25 Supporting	rat	14	n/a	n/a	n/a	n/a	n/a	1 500 mg/kg bw/day	n/a	n/a	n/a
26 Supporting	monkey	21	n/a	n/a	n/a	n/a	n/a	500 mg/kg bw/day	n/a	n/a	Males only
27 Supporting	rat	90	n/a	n/a	n/a	n/a	n/a	10 mg/kg bw/day	n/a	Liver weight ↑	Males only
28 Supporting	rat	3, 7, 14, 28	n/a	n/a	n/a	n/a	n/a	n/a	50 mg/kg bw/day	Number of peroxisomes ↑	n/a
29 Supporting	mouse	728	n/a	n/a	n/a	n/a	n/a	n/a	3 000 ppm	Carcinogenicity	Duration
30 Supporting	monkey	14	n/a	n/a	n/a	n/a	n/a	n/a	2 000 mg/kg bw/day	Relative kidney weights ↓ in females, hepatic catalase activity ↑ in males.	n/a
31 Supporting	rat	7, 21	n/a	n/a	n/a	n/a	n/a	n/a	2 500 mg/kg bw/day	n/a	n/a
32 Supporting	rat	90	Histopathology	n/a	Histopathology	n/a	n/a	6 300 ppm (320 mg/kg bw/d)	n/a	Testes atrophy	n/a
33 Supporting	rat	28	n/a	n/a	n/a	n/a	n/a	n/a	50 mg/kg bw/day (male)	Liver weight ↑ and enzyme activity	n/a
34 Supporting	monkey	14	n/a	n/a	n/a	n/a	n/a	500 mg/kg bw/day	n/a	n/a	Males only
35 Other	rat	n/a	n/a	n/a	n/a	n/a	n/a	n/a	n/a	n/a	Duration (lifetime)
36 Other	Dog	365	Histopathology	n/a	n/a	n/a	n/a	n/a	n/a	n/a	Duration
37 Other	Dog	365	Histopathology	n/a	n/a	n/a	n/a	0.06 ml/kg/day	n/a	n/a	Duration
38 Other	mouse	28	Histopathology (absence of corpora lutea)	n/a	n/a	n/a	n/a	1 000 ppm	n/a	Liver weight ↑, kidney weight ↓, body weight ↓	No effect level (in mg/kg bw/day)
39 Other	rat	7	n/a	n/a	n/a	n/a	n/a	n/a	n/a	n/a	Males only
40 Other	rat	21	n/a	n/a	n/a	n/a	n/a	n/a	n/a	n/a	Males only
41 Other	guinea pig	365	n/a	n/a	n/a	n/a	n/a	n/a	0.04 % in diet	n/a	Duration
42 Other	guinea pig	365	n/a	n/a	n/a	n/a	n/a	n/a	n/a	n/a	Duration
43 Other	rat	28	n/a	n/a	n/a	n/a	n/a	n/a	0.67%	Liver weight ↑, number of peroxisome proliferation and induction of enzymes↑	No effect level (in mg/kg bw/day)
44 Other	rat	90	n/a	n/a	n/a	n/a	n/a	n/a	1 000 ppm (63 mg/kg bw/day males and 73 mg/kg bw/day females)	Liver weight ↑	n/a
45 Other	mouse	10	n/a	n/a	n/a	n/a	n/a	n/a	n/a	n/a	Males only
46 Other	cat	60	n/a	n/a	n/a	n/a	n/a	n/a	n/a	n/a	No effect level
47 Other	rat	28	n/a	n/a	n/a	n/a	n/a	6 000 ppm	n/a	Testicular toxicity	Males only
48 Other	rabbit	52	n/a	n/a	n/a	n/a	n/a	n/a	n/a	n/a	No effect level

49 Other	rat	28	Histopathology	n/a	n/a	n/a	n/a	n/a	0.2%	Liver weight ↑, body weight ↓	No effect level (in mg/kg bw/day)
50 Other	rat	21	n/a	n/a	n/a	n/a	n/a	n/a	n/a	n/a	Males only
51 Other	mouse	5	n/a	n/a	n/a	n/a	n/a	n/a	n/a	n/a	Males only
52 Other	rat	n/a	Histopathology	n/a	n/a	n/a	n/a	0.06 mg/kg bw/day	0.2 mg/kg bw/day	n/a	Duration (multigenerational study)
53 Other	rat	n/a	n/a	n/a	n/a	n/a	n/a	n/a	n/a	n/a	Duration and males only
54 Other	mouse	14	n/a	n/a	n/a	n/a	n/a	n/a	n/a	n/a	No effect level
55 Other	dog	98	n/a	n/a	n/a	n/a	n/a	n/a	n/a	n/a	No effect level
56 Other	Rat	14	n/a	n/a	n/a	n/a	n/a	n/a	n/a	n/a	Males only
**57 Other**	**rat**	**3**	**n/a**	**n/a**	**n/a**	**n/a**	**n/a**	**50 - 80 mg/kg bw/day**	**n/a**	**Liver and kidney weight ↑, body weight ↓**	**n/a**
58 Other	rat	21	n/a	n/a	n/a	n/a	n/a	n/a	n/a	n/a	Males only
59 Other	rat	14	n/a	n/a	n/a	n/a	n/a	n/a	1 000 mg/kg bw/day	Liver weight ↑	Males only
60 Other	rat	112	n/a	n/a	n/a	n/a	n/a	n/a	n/a	n/a	Males only
61 Other	rat	14	n/a	n/a	n/a	n/a	n/a	250 mg/kg bw/day	n/a	Liver weight ↑	Males only
62 Other	rat	28	n/a	n/a	n/a	n/a	n/a	n/a	n/a	n/a	Males only
**63 Other**	**monkey**	**90**	**Pathology**	**n/a**	**Pathology**	**n/a**		**500 mg/kg bw/day**	**n/a**	**Body weight gain ↓**	**n/a**
64 Other	rat	3-60 days	n/a	n/a	n/a	n/a	n/a	n/a	n/a	n/a	Males only
65 Other	rat	90	n/a	n/a	n/a	n/a	n/a	n/a	0.9 mg/kg bw/day	Effect on creatinine clearance	Males only
66 Other	mouse	8	n/a	n/a	n/a	n/a	n/a	n/a	n/a	n/a	No effect level
**67 Other**	**rat**	**7, 21**	**n/a**	**n/a**	**n/a**	**n/a**	**n/a**	**n/a**	**0.1% (80 mg/kg bw/day)**	**Liver weight ↑**	**n/a**
68 Other	rat	7	n/a	n/a	n/a	n/a	n/a	n/a	2% in diet	Liver and kidney weight ↑	Males only
69 Other	rat	30	n/a	n/a	n/a	n/a	n/a	n/a	10 mg/kg bw/day	Decreased serum testosterone levels; increased reactive oxygen species formation, impaired insulin signal transduction and glucose uptake in skeletal muscle	Males only
70 Other	rat	28	n/a	n/a	n/a	n/a	n/a	n/a	n/a	n/a	Males only
71 Other	rat	14	n/a	n/a	n/a	n/a	n/a	n/a	10 000 ppm	n/a	Males only
72 Other	rat	21	n/a	n/a	n/a	n/a	n/a	n/a	n/a	n/a	Males only

73 Other	rat	1-365	n/a	n/a	n/a	n/a	n/a	n/a	n/a	n/a	Males only
74 Other	rat	14	n/a	n/a	n/a	n/a	n/a	n/a	n/a	n/a	No effect level
75 Other	rat	7	n/a	n/a	n/a	n/a	n/a	n/a	2 % in diet	Liver and kidney weight ↑	Males only
76 Other	rat	28	microscopical examination	n/a	n/a	n/a	n/a	n/a	0.2%	Liver weight ↑, body weight ↓	No effect level (in mg/kg bw/day)
77 Other	mouse	14	n/a	n/a	n/a	n/a	n/a	n/a	n/a	n/a	No effect level

Table S3. All identified studies in ECHA's CHEM database where repeated dose toxicity of DEHP was investigated *in vivo*. Red font denotes excluded studies, according to the exclusion criteria in the last column. Black font denotes included studies (also listed in Table S1).

Data set	Species	Duration (days)	ovar*	follic*	uter*	proges*	(o)estro*	NOAEL	LOAEL	NOAEL/LOAEL finding	Exclusion criteria
Key 1	Rat	728	n/a	n/a	gross pathology	n/a	n/a	500 ppm (28.9 mg/kg bw/day [males] and 36.1 mg/kg bw/day [females])	n/a	Liver: ↑ weight (males) and peroxisome proliferation at 500 ppm; kidney: ↑ weight from 2 500 ppm	Duration
Key 2	Mouse	728	Histopathology	n/a	Histopathology, weight	n/a	n/a	100 ppm (19.2 mg/kg bw/day [males] and 23.8 mg/kg bw/day [females])	n/a	Liver: peroxisome proliferation and ↑ weight (males) from 500 ppm; ↑ weight, adenomas and carcinomas (both sexes) from 1 500 ppm	Duration
Key 3	Rat	90	Gross- and histopathology	n/a	Gross- and histopathology	n/a	n/a	50 ppm (3.7 mg/kg bw/day)	n/a	Testes: mild to moderate Sertoli cell vacuolation from 500 ppm	n/a
Key 4	Monkey	91	Pathology	n/a	Pathology	n/a	n/a	500 mg/kg bw/day	n/a	Decreased body weight gain at 2 500 mg/kg bw/day	n/a
5 Supporting	Rat	28	n/a	n/a	n/a	n/a	n/a	51.7 mg/kg bw/day	n/a	Induction of palmitoyl-CoA oxidation	Males only
6 Supporting	Rat	730	n/a	n/a	n/a	n/a	n/a	n/a	n/a	n/a	Males only
7 Supporting	Mouse	91	Pathology	n/a	Pathology	n/a	n/a	800 ppm (100 mg/kg bw/day)	n/a	↓bwg from 3 100 ppm (males) and from 1 600 ppm (females)	n/a
8 Supporting	Monkey	14	n/a	n/a	n/a	n/a	n/a	500 mg/kg bw/day	n/a	n/a	Males only
9 Supporting	Rat	730	n/a	n/a	n/a	n/a	n/a	n/a	0.02% in the diet (7 mg/kg bw/day)	Testes atrophy and inhibition of spermatogenesis	Males only
10 Supporting	Rat	728	n/a	n/a	n/a	n/a	n/a	n/a	6 000 ppm	No NOAEL determined	Duration
11 Supporting	Rat	1-365	n/a	n/a	n/a	n/a	n/a	n/a	n/a	n/a	Males only
12 Supporting	Mouse	728	n/a	n/a	n/a	n/a	n/a	3 000 ppm	n/a	The LOAEL for carcinogenicity has been determined at 3 000 ppm	Duration
13 Supporting	Rat	90	Pathology	n/a	Pathology	n/a	n/a	6 300 ppm (320 mg/kg bw/day)	n/a	↓bwg at 25 000 ppm. Testes: atrophy from 12 500 ppm	n/a

Table S4. All included studies from both ECHA's registered factsheet and CHEM databases where reproductive toxicity of DEHP was investigated *in vivo*. Unless indicated otherwise with ”ECHA CHEM”, most included studies were retrieved from ECHA registered factsheet database.

Data set	Species	Duration (days)	ovar*	follic*	uter*	proges*	(o)estrou*	NOAEL	LOAEL	NOAEL/LOAEL finding
3 Supporting	mouse	98	n/a	n/a	n/a	n/a	Vaginal cytology	n/a	P0: 3 000 ppm (150 mg/kg bw7day)	Reduction in epididymal and testicular weights, sperm motility, sperm concentration; increased number of abnormal sperm cells were observed at the only dose tested.
12 Supporting	mouse	2, 4, 7, 14, 21, 28 and 30	n/a	n/a	n/a	n/a	n/a	P0: 140 - 493 mg/kg bw/day	n/a	n/a
15 Supporting	rat	90	histopathology	n/a	histopathology	n/a	n/a	3.7 mg/kg bw/day	n/a	High incidence of minimal to mild Sertoli cell vacuolation at 500 ppm
18 Supporting	rat	90	weight and histopathology	n/a	histopathology	n/a	n/a	1 060 mg/kg bw/day (110 mg/kg bw/day (development))	n/a	↑ relative liver weight in F0 females from 1,000 ppm
19 Supporting	mouse	7+98	weight (F0)	n/a	weight (F0)	n/a	n/a	14 mg/kg bw/day	141 mg/kg bw/day	Reductions in litter size and proportions of pairs having litters.
20 Supporting	mouse	17	weight and histopathology	n/a	weight and histopathology	n/a	n/a	≥ 95 mg/kg bw/day	n/a	n/a
22 Supporting	monkey	91	n/a	n/a	n/a	n/a	n/a	> 2500 mg/kg bw/day	n/a	n/a
26 Supporting	rat	90	n/a	n/a	n/a	n/a	n/a	320 mg/kg bw/day (males)	n/a	Testis atrophy
28 Supporting	Rat	10	Weight, steroidogenic profile,	n/a	n/a	n/a	The stage of estrus determined before euthanized	n/a	1 500 mg/kg bw/day	Alterations of the oestrus cycle and concentration changes of testosterone and oestradiol in rats in dioestrus.
29 Supporting	rat	1-12d	Morphological evaluation	Classified by size and morphology	Histopathology	Levels determined	Cyclicity determined and studied	n/a	2 000 mg/kg bw/day	Prolonged oestrus cycles, suppressed or delayed ovulation
31 Supporting	rat	45	n/a	n/a	n/a	n/a	n/a	300 ppm (55 – 58 mg/kg bw/day)	n/a	n/a
35 Other	rat	22 (doses on d 1, 5, 10)	yes	n/a	n/a	n/a	n/a	n/a	5 ml/kg (4 900 mg/kg bw/day)	Testes and ovary enzyme activity
7 Supporting ECHA CHEM	Mouse	17	Weight, histopathology	n/a	Weight, histopathology	n/a	Estrous cyclicity of F1 females	≥ 95 mg/kg bw/day	n/a	n/a
13 Supporting ECHA CHEM	Rat	n/a	Weight, steroidogenic profile,	n/a	n/a	n/a	The stage of estrus determined before euthanized	n/a	1 500 mg/kg bw/day	Alterations of the oestrus cycle and concentration changes of testosterone and oestradiol in rats in dioestrus.
14 Supporting ECHA CHEM	Rat	1-12d	Morphological evaluation	Classified by size and morphology	Histopathology	Levels determined	Cyclicity determined and studied	n/a	2 000 mg/kg bw/day	Prolonged oestrus cycles, suppressed or delayed ovulation

Table S5. All identified studies in ECHA's registered factsheet database where reproductive toxicity of DEHP was investigated *in vivo*. Red font denotes excluded studies, according to the exclusion criteria in the last column. Black font denotes included studies (also listed in Table S4).

Data set	Species	Duration (days)	ovar*	follic*	uter*	proges*	(o)estrou*	NOAEL	LOAEL	NOAEL/LOAEL finding	Exclusion criteria
1 Key	rat	n/a, three-generation study	Organ weight and histopathology	n/a	Organ weight and histopathology	n/a	Assessed in (FO), (F1c), (F2c) animals upon completion of the lactation phase	100 ppm (3 – 5 mg/kg bw/day)	n/a	Developmental toxicity	Duration
2 Key	rat	n/a, three-generation study	Organ weight and histopathology	n/a	Organ weight and histopathology	n/a	Assessed in (FO), (F1c), (F2c) animals upon completion of the lactation phase	100 ppm (3 – 5 mg/kg bw/day)	n/a	Developmental toxicity	Duration
**3 Supporting**	**mouse**	**98**	**n/a**	**n/a**	**n/a**	**n/a**	**Vaginal cytology**	**n/a**	**P0: 3 000 ppm (150 mg/kg bw7day)**	**Reduction in epididymal and testicular weights, sperm motility and, sperm concentration; increased number of abnormal sperm cells were observed at the only dose tested.**	**n/a**
4 Supporting	rat	60	n/a	n/a	n/a	n/a	n/a	1 250 ppm (9 mg/kg bw/day)	n/a	Total body, testis, epididymis, and prostate weights ↓	Males only
5 Supporting	rat	714	n/a	n/a	n/a	n/a	n/a	n/a	0.02 % in diet (7 mg/kg bw/day)	Testes: atrophy and inhibition of spermatogenesis	Males only and duration
6 Supporting	mouse	21-28	Histology, weight, lysosomal and ATP activity, as well as RNA, DNA and protein content	Assessed	n/a	n/a	n/a	n/a	1 ml/kg	Reduction in the incidence of pregnancies	No effect level (in mg/kg bw/day)
7 Supporting	rat	2	n/a	n/a	n/a	n/a	n/a	20 mg/kg bw/day	n/a	Altered gon/acyte morphology and decreased Sertoli cell proliferation	Males only
8 Supporting	rat	15	n/a	n/a	n/a	n/a	n/a	n/a	250 mg/kg bw/day	Sperm count ↓	Males only
9 Supporting	rat	21	n/a	n/a	n/a	n/a	n/a	n/a	300 mg/kg bw/day	Testicular toxicity	Males only
10 Supporting	monkey	455	Organ weight, gross pathology and histopathology	n/a	Organ weight, gross pathology and histopathology	n/a	n/a	100 mg/kg bw/day	n/a	Increased ovarian and uterine weights and elevated blood estradiol levels were observed in higher dosage groups	Duration
11 Supporting	rat	n/a	n/a	n/a	n/a	n/a	n/a	n/a	100 mg/kg bw/day.	Serum concentration of testosterone ↓	Males only and in utero exposure
**12 Supporting**	**mouse**	**2, 4, 7, 14, 21, 28 and 30**	**n/a**	**n/a**	**n/a**	**n/a**	**n/a**	**P0: 140 - 493 mg/kg bw/day**	**n/a**	**n/a**	**n/a**
13 Supporting	rat	721	n/a	n/a	n/a	n/a	n/a	n/a	322 mg/kg bw/day	testes: seminiferous tubular degeneration at 6 000 and 12 000 ppm	Duration
14 Supporting	rat	721	n/a	n/a	n/a	n/a	n/a	500 ppm (28.9 mg/kg bw/day)	n/a	Testicular toxicity	Duration
**15 Supporting**	**rat**	**90**	**histopathology**	**n/a**	**histopathology**	**n/a**	**n/a**	**3.7 mg/kg bw/day**	**n/a**	**High incidence of minimal to mild Sertoli cell vacuolation at 500 ppm**	**n/a**
16 Supporting	rat	n/a, one-generation study	n/a	n/a	n/a	n/a	n/a	1.215 mg/kg bw/day	5 mg/kg bw/day	Reduced daily sperm production	In utero/lactation exposure
17 Supporting	rat	n/a, two-generation study	n/a	number of corpora lutea	no. of uterine implants examined	n/a	n/a	100 mg/kg bw/day (males)	n/a	Testicular toxicity	Males only
**18 Supporting**	**rat**	**90**	**weight and histopathology**	**n/a**	**histopathology**	**n/a**	**n/a**	**1060 mg/kg bw/day (110 mg/kg bw/day (development))**	**n/a**	**↑ relative liver weight in F0 females from 1 000 ppm**	**n/a**

19 Supporting	mouse	7+98	weight (F0)	n/a	weight (F0)	n/a	n/a	14 mg/kg bw/day	141 mg/kg bw/day	Reductions in litter size and proportions of pairs having litters.	n/a
20 Supporting	mouse	17	weight and histopathology	n/a	weight and histopathology	n/a	n/a	95 mg/kg bw/day	n/a	n/a	n/a
21 Supporting	rat	28	n/a	n/a	n/a	n/a	n/a	1 mg/L	n/a	Testicular toxicity	Males only
22 Supporting	monkey	91	n/a	n/a	n/a	n/a	n/a	> 2500 mg/kg bw/d	n/a	n/a	n/a
23 Supporting	mouse	728	n/a	n/a	n/a	n/a	n/a	500 ppm (98.5mg/kg bw/day)	n/a	Testis: from 1 500 ppm ↓ weight, ↑ incidence and severity of bilateral hypospermia; epididymis: from 1 500 ppm ↑ immature or abnormal sperm forms and hypospermia; changes in testes partially reversible)	Males only and duration
24 Supporting	mouse	728	n/a	n/a	n/a	n/a	n/a	672 mg/kg bw/day	n/a	Testes: seminiferous tubular degeneration and testicular atrophy at 6 000 ppm	Duration
25 Supporting	rat	21	n/a	n/a	n/a	n/a	n/a	P0: 60 mg/kg bw/day	n/a	Testicular toxicity	Males only
26 Supporting	rat	90	n/a	n/a	n/a	n/a	n/a	320 mg/kg bw/day (males)	n/a	Testis atrophy	n/a
27 Supporting	rat	n/a, two-generation study	weight and histopathology	ovarian counts examined	weight and histopathology		Estrous cycle length and normality were evaluated daily for all F0 and F1 female parental rats	n/a	1 000 ppm (113 mg/kg and day)	Sertoli cell vacuolations recorded in the F1 offspring generation	Duration (multigenerational study)
28 Supporting	Rat	10	Weight, steroidogenic profile,	n/a	n/a	n/a	The stage of estrus determined before euthanized	n/a	1 500 mg/kg bw/day	Alterations of the oestrus cycle and concentration changes of testosterone and oestradiol in rats in dioestrus.	n/a
29 Supporting	rat	1-12	Histopathology	Follicles classified	histopathology	Progesterone levels determined	vaginal cytology and vaginal histology	n/a	2 000mg/kg bw/d (cyclicity)	Prolonged oestrus cycles, suppressed or delayed ovulation	n/a
30 Supporting	rat	119	n/a	n/a	Histopathology	n/a	n/a	n/a	0.2 % (143 mg/kg bw/day)	Liver: ↑absw and relw	Duration
31 Supporting	rat	45	n/a	n/a	n/a	n/a	n/a	300 ppm (55 – 58 mg/kg bw/d)	n/a	n/a	n/a
32 Other	rat	15	n/a	n/a	n/a	n/a	n/a	n/a	500 mg/kg bw/day	n/a	Males only
33 Other	ferret	426	n/a	n/a	n/a	n/a	n/a	n/a	1 200 mg/kg bw/day	n/a	Males only and duration
34 Other	rat	28	n/a	n/a	n/a	n/a	n/a	6 000 ppm	n/a	Testicular toxicity	Males only
35 Other	rat	22 (doses on d 1, 5, 10)	yes	n/a	n/a	n/a	n/a	n/a	5 ml/kg (4 900 mg/kg bw/d)	Testes and ovary enzyme activity	n/a
36 Other	rat	6	n/a	n/a	n/a	n/a	n/a	5 mg/kg bw/day	n/a	n/a	Males only
37 Other	rat	7	n/a	n/a	n/a	n/a	n/a	n/a	2 000 mg/kg bw/day	n/a	Males only
38 Other	mouse	7	n/a	n/a	n/a	n/a	n/a	n/a	1 000 mg/kg bw/day	Reduced fertility reduced. Sperm density and sperm motility significantly reduced	Males only
39 Other	rat	42	n/a	n/a	n/a	n/a	n/a	n/a	0.033 mg/L in drinking water	n/a	No effect level (in mg/kg bw/day)
40 Other	hamster	9	n/a	n/a	n/a	n/a	n/a	n/a	4 200 mg/kg bw/day	n/a	Males only
41 Other	rat	14	n/a	n/a	n/a	n/a	n/a	n/a	2 000 mg/kg bw/day	Testicular toxicity	Males only
42 Other	mouse	On days 1, 5 and 10	n/a	n/a	n/a	n/a	n/a	n/a	1 ml/kg bw	Reduction in the incidence of pregnancy	Males only

Table S6. All identified studies in ECHA's CHEM database where reproductive toxicity of DEHP was investigated *in vivo*. Red font denotes excluded studies, according to the exclusion criteria in the last column. Black font denotes included studies (also listed in Table S4).

Data set	Species	Duration (days)	ovar*	follic*	uter*	proges*	(o)estro*	NOAEL	LOAEL	NOAEL/LOAEL finding	Exclusion criteria
1 Key	Rat	n/a, three-generation study	Weight, histopathology	n/a	Weight, histopathology	n/a	Oestrous cyclicity assessed on (FO), (F1c), (F2c): Upon completion of the lactation phase	P0: 300 ppm (23 mg DEHP/kg bw/day) body weight and weight gain, 1 000 ppm (77 mg DEHP/kg bw/day)	n/a	Increased liver: 1 000 ppm	Duration
2 Supporting	Rat	1	n/a	n/a	n/a	n/a	n/a	20 mg/kg bw/day	n/a	n/a	Males only
3 Supporting	Rat	21	n/a	n/a	n/a	n/a	n/a		300 mg/kg/day	Testicular changes	Males only
4 Supporting	Rat	728	n/a	n/a	n/a	n/a	n/a	-	322 mg/kg bw/day	Testes: seminiferous tubular degeneration at 6 000 and 12 000 ppm	Duration
5 Supporting	Monkey	455	Weight, gross+histopathology	n/a	Weight, gross+histopathology	n/a	n/a	100 mg/kg bw/day	n/a	Increased ovarian and uterine weights and elevated blood estradiol levels were observed in higher dosage groups	Duration
6 Supporting	Rat	n/a, one-generation study	n/a	n/a	n/a	n/a	n/a	1.215 mg/kg bw/day	n/a	Reduced daily sperm production and has the potential to induce reproductive tract abnormalities	Males only
**7 Supporting**	**Mouse**	**17**	**Weight, histopathology**	**n/a**	**Weight, histopathology**	**n/a**	**Oestrous cyclicity of F1 females**	**≥ 95 mg/kg bw/day**	**n/a**	**n/a**	**n/a**
8 Supporting	Rat	28	n/a	n/a	n/a	n/a	n/a	360 mg/kg	n/a	n/a	Males only
9 Supporting	Monkey	91	n/a	n/a	n/a	n/a	n/a	>2 500 mg/kg bw/d	n/a	n/a	Males only
10 Supporting	Mouse	728	n/a	n/a	n/a	n/a	n/a	672 mg/kg bw/day (males)	n/a	Seminiferous tubular degeneration and testicular atrophy at 6 000 ppm	Duration
11 Supporting	Rat	21	n/a	n/a	n/a	n/a	n/a	60 mg/kg bw/day (males)	n/a	Testicular toxicity	Males only
12 Supporting	Rat	n/a, two-generation study	Weight, histopathology	n/a	Weight, histopathology	n/a	Length and normality were evaluated daily for all F0 and F1 parental rats	P0: 3 000 ppm (339.5 mg/kg bw/day)	F1: 1 000 ppm, 113 mg/kg and day	Focal tubular atrophy	Duration
**13 Supporting**	**Rat**	**10**	**Weight, steroidogenic profile,**	**n/a**	**n/a**	**n/a**	**The stage of estrus determined before euthanized**	**n/a**	**1 500 mg/kg bw/day**	**Alterations of the oestrus cycle and concentration changes of testosterone and oestradiol in rats in dioestrus.**	**n/a**
**14 Supporting**	**Rat**	**12**	**Morphological evaluation**	**Classified by size and morphology**	**Histopathology**	**Levels determined**	**Cyclicity determined and studied**	**n/a**	**2 000 mg/kg bw/day**	**Prolonged oestrus cycles, suppressed or delayed ovulation**	**n/a**
